# Pre-Implantation Bovine Embryo Evaluation—From Optics to Omics and Beyond

**DOI:** 10.3390/ani13132102

**Published:** 2023-06-24

**Authors:** R. A. Chanaka Rabel, Paula V. Marchioretto, Elizabeth A. Bangert, Kenneth Wilson, Derek J. Milner, Matthew B. Wheeler

**Affiliations:** 1 Department of Animal Sciences, University of Illinois at Urbana-Champaign, Urbana, IL 61801, USA; rrabel2@illinois.edu (R.A.C.R.); paulav@illinois.edu (P.V.M.); eb21@illinois.edu (E.A.B.); kwilson8@illinois.edu (K.W.); dmilner@illinois.edu (D.J.M.); 2 Carl R. Woese Institute for Genomic Biology, University of Illinois at Urbana-Champaign, Urbana, IL 61801, USA; 3 Department of Bioengineering, University of Illinois at Urbana-Champaign, Urbana, IL 61801, USA; 4 Department of Biomedical and Translational Sciences, Carle-Illinois College of Medicine, University of Illinois at Urbana-Champaign, Urbana, IL 61801, USA

**Keywords:** bovine embryo evaluation, in vitro production, in vivo derived embryos, in vitro fertilization, bovine embryo grading, pre-implantation embryo quality, dairy cattle blastocyst morphological analysis, beef cattle embryo staging, bovine embryo transfer, assisted reproductive technologies

## Abstract

**Simple Summary:**

With conventional breeding practices, even the best dairy/beef cows will likely produce no more than half-a-dozen calves in their lifetimes; a crime, would you not agree? In contrast, with embryo transfer technologies, a cow can pass her genetics to hundreds of calves through surrogate cows. As such, embryo transfer, particularly involving embryos produced by in vitro fertilization, also known as IVF, is being widely adopted. However, pregnancy rates from the transfer of IVF embryos remain unimpressive. Prior to transfer, embryos are evaluated under a microscope, and only high-quality embryos are transferred. Unfortunately, microscopy cannot accurately distinguish between high-quality and low-quality embryos. Many embryo evaluation methodologies have been tested to date to find a replacement for microscopy-based embryo evaluation, e.g., advanced microscopic systems and the evaluation of genetics and secreted proteins/molecules. The good news: many of them can accurately separate the wheat from the chaff. The bad news: many of them are expensive, kill embryos during evaluation, are too complicated, and/or are laborious and thus useless in the frontline, i.e., thousands of embryo production/transfer facilities. This article reviews the most common (and not so common) techniques that have been tested to date and provides insights about those that have the highest potential to replace microscopy-based embryo evaluation *in*-*field* applications.

**Abstract:**

Approximately 80% of the ~1.5 million bovine embryos transferred in 2021 were in vitro produced. However, only ~27% of the transferred IVP embryos will result in live births. The ~73% pregnancy failures are partly due to transferring poor-quality embryos, a result of erroneous stereomicroscopy-based morphological evaluation, the current method of choice for pre-transfer embryo evaluation. Numerous microscopic (e.g., differential interference contrast, electron, fluorescent, time-lapse, and artificial-intelligence-based microscopy) and non-microscopic (e.g., genomics, transcriptomics, epigenomics, proteomics, metabolomics, and nuclear magnetic resonance) methodologies have been tested to find an embryo evaluation technique that is superior to morphologic evaluation. Many of these research tools can accurately determine embryo quality/viability; however, most are invasive, expensive, laborious, technically sophisticated, and/or time-consuming, making them futile in the context of *in-field* embryo evaluation. However accurate they may be, using complex methods, such as RNA sequencing, SNP chips, mass spectrometry, and multiphoton microscopy, at thousands of embryo production/collection facilities is impractical. Therefore, future research is warranted to innovate *field-friendly*, simple *benchtop tests* using findings already available, particularly from omics-based research methodologies. Time-lapse monitoring and artificial-intelligence-based automated image analysis also have the potential for accurate embryo evaluation; however, further research is warranted to innovate economically feasible options for *in-field* applications.

## 1. Introduction

**Figure 1:**
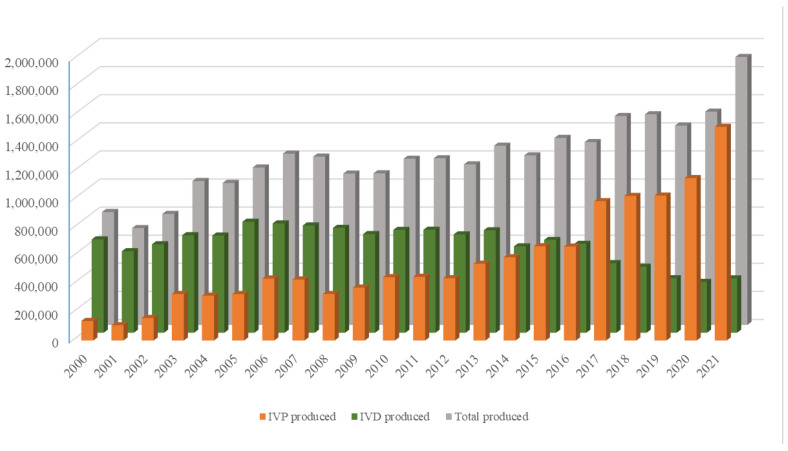
Annual production of IVP (orange bars), IVD (green bars), and total number (gray bars) of bovine embryos. Compiled from 2001–2022 annual Data Retrieval Committee Reports of the International Embryo Transfer Society.

**Figure 2:**
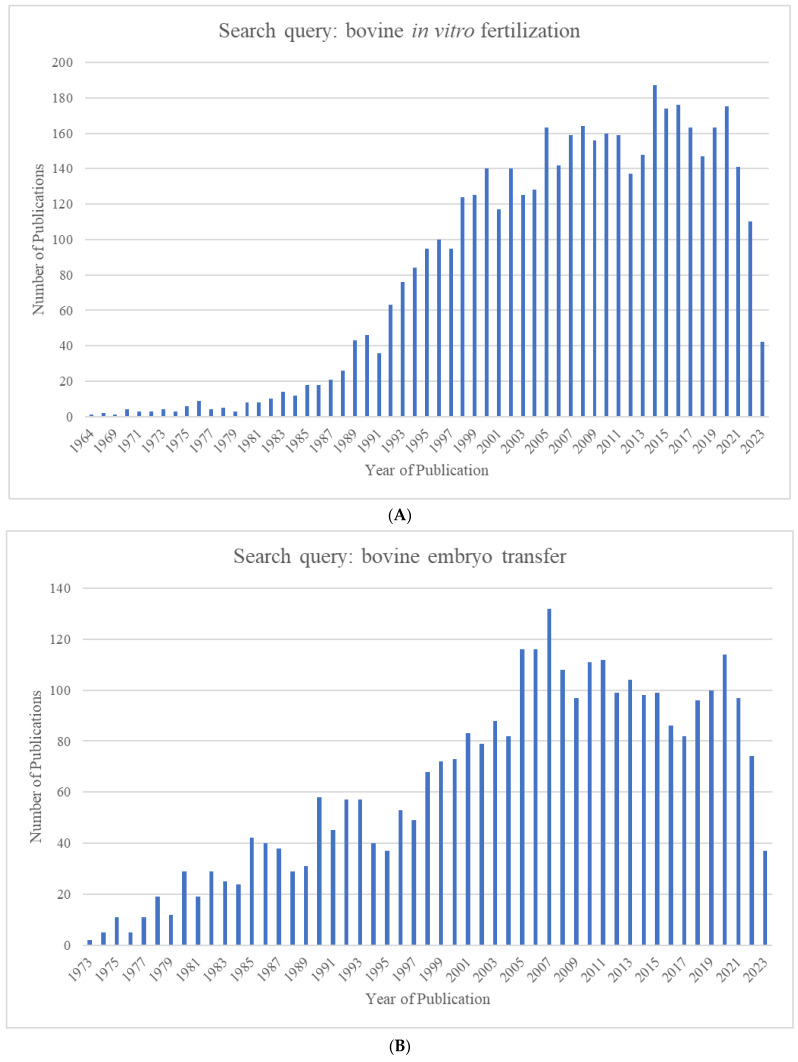

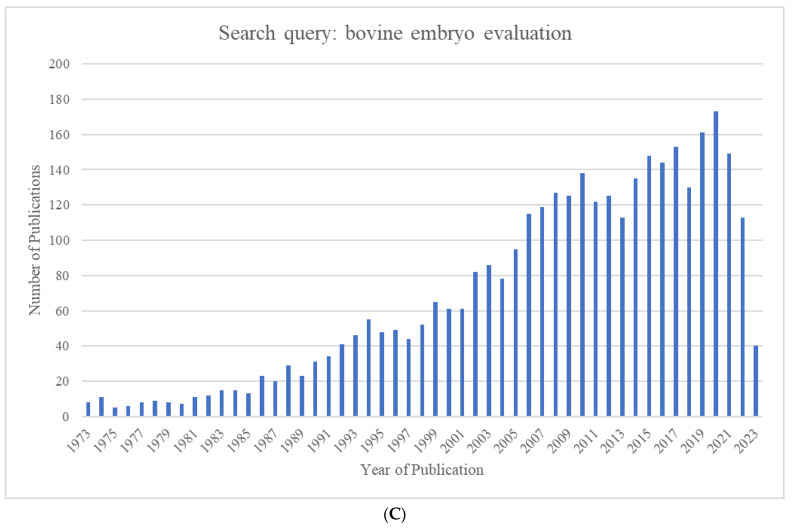
Number of publications identified in Pubmed.gov by year for the search queries (**A**) "bovine in vitro fertilization” (1964–2023), (**B**) “bovine embryo transfer” (1973–2023), and (**C**) “bovine embryo evaluation” (1973–2023).

The growing world population has created an ever-increasing demand for animal proteins. The beef and dairy industries play tremendous roles in this regard, annually contributing >72 million metric tons of meat (21% of global meat production) and >500 million metric tons of milk to the global market. *Reproduction* is a major driver of the bovine meat and milk *production* industries. As such, Assisted Reproductive Technologies (ARTs) such as in vitro embryo production (IVP), in vivo derived embryo production (IVD), and embryo transfer (ET) have gained enormous importance in the beef and dairy cattle industries [1]. As illustrated in Figure 1, the annual production of transferrable bovine embryos has nearly doubled within the 12-year period from 2009 to 2021. What is even more striking is the shift of embryo production from IVD to IVP, with embryo production by IVP quadrupling over the same period. In 2021, over 1.5 million transferrable IVP embryos were produced (Figure 1), of which nearly 80% (~1.2 million) were transferred [2]. Parallel to this rapid expansion of IVP, the interest from the scientific community and, in turn, research publications have also risen many-fold over the last few decades (Figure 2A–C).

It is commonly accepted that the developmental competence, quality, and cryo-survival of IVP embryos are overall lower than those of IVD embryos. Further, pregnancy rates (PRs) resulting from the transfer of IVP embryos are lower than those from the transfer of quality- and stage-matched IVD embryos reviewed by [3,4,5,6]. For both IVP and IVD embryos, it is well established that the transfer of higher-quality blastocysts results in higher PRs compared to the transfer of lower-quality blastocysts [7,8,9]. Therefore, if embryos can be accurately evaluated and the high-quality ones could be selected for ET, the commercial value of selected embryos would increase, with a subsequent decrease in the number of recipients and an overall improvement in the efficiency of ET [10]. Therefore, one of the most important factors associated with the successful application of ARTs is *embryo evaluation* prior to freezing or transfer to recipients [11,12]. At present, the most widely used method for determining embryo quality is morphological evaluation using stereomicroscopy [6]. However, it is considered to have poor accuracy and poor reproducibility and to be biased by the subjectivity of the evaluator, i.e., intra- and inter-observer variability [1,5].

Together, the current trend favoring IVP and the limitations of current morphology-based embryo evaluation methods demand the in-depth study of pre-implantation bovine embryos (i) to better understand the physiology of embryo development and (ii) to discover embryo viability/quality markers that can be used for the accurate prediction of post-transfer embryo survival and the pregnancy outcome. As a result, a wide variety of methodologies, ranging from optics to omics-based methodologies to gene editing and everything else in between, have been used to evaluate and analyze bovine embryos.

The aim of this review is to provide an overview of the various embryo evaluation methodologies used not only during the post-IVP era but also starting from 1931, the first available record of the microscopic anatomy of pre-implantation bovine embryos from nearly a century ago [13]. Further, we also provide insights about embryo evaluation methodologies that have the highest potential for adoption at the *ground level* as a replacement for conventional stereomicroscopy-based morphological embryo evaluation.

We conclude that sophisticated embryo evaluation tools such as omics-based methodologies are unfit to be *directly* used for *in-field* embryo evaluation. However, findings from these methodologies can be applied *indirectly* for *in-field* embryo evaluation if *field-friendly*, simple, and affordable *benchtop tests* are developed. Further, findings from time-lapse monitoring (TLM) and artificial-intelligence-based automated image analysis look very promising. With further improvements and the innovation of economically affordable options, they may be directly used for *in-field* applications.

## 2. Evaluation of Pre-Implantation Bovine Embryos

Embryo evaluation is two-fold and is typically performed prior to cryopreservation. They are evaluated for the *developmental stage (grade)* and *quality*. Currently, only embryos satisfying minimum criteria in both these facets are selected for freezing or direct transfer.

### 2.1. Evaluation for Embryo Quality

The microscopic anatomy of the bovine embryo was first described in 1931 for a *two-celled ovum* [13], and subsequent stages up to the blastocyst stage were characterized in 1946 [14]. However, it was not until the mid-1970s, when bovine embryo transfer, carried out at an experimental scale thus far, was transformed into a commercial-scale venture [15], that the first attempts at embryo evaluation were made [16]. The authors rated 8–12-celled embryos and morulae on a scale of 1 to 5 (Table 1) “*on the basis of compactness, symmetry and density of the blastomeres*”. They observed that pregnancy rates resulting from the transfer of morulae were higher than those resulting from the transfer of 8–12-celled embryos, and that the highest pregnancy rates were obtained by transferring morulae of the highest quality, i.e., a rating of 5. However, the authors admitted that *“This rating, by its very nature, was subjective and hence any rigid analysis of the results would not be truly scientific”*.

A set of relatively less subjective criteria were used in a 1978 report that categorized embryos into four classes, namely, excellent, good, fair, and poor ([17]; Table 1). They observed that, upon transfer, embryos categorized as “excellent” and “good” gave the highest pregnancy rates (PRs), while those categorized as “poor” gave the lowest PRs. A 1981 study adopted a classification system where embryos were given ratings of 2, 3, or 4 ([18]; Table 1). Similar to previous studies, embryos with a higher rating gave higher pregnancy rates compared to embryos with lower ratings. A 1983 study ([7]; Table 1) used the same “excellent”, “good”, “fair”, and “poor” classification system used by Elsden, Nelson, 1978 [17], but with slightly modified defining criteria. Consistent with previous studies, excellent and good embryos gave the best pregnancy rates. They went on to conclude that embryo quality, but not the stage of development (16-cell through hatched blastocysts), is the most accurate predictor of an embryo’s competency to establish a pregnancy. This finding contradicted the previous observations of Shea, Hines, 1976 [16], and Newcomb and Rowson, 1975 [19], where the authors observed higher PRs when transferring morulae compared to 8–12-celled embryos and higher PRs when transferring 5–7-day-old embryos compared to <5-day-old embryos, respectively. These observations highlight the need to devise strategies for embryo grading, as very early stage embryos with as few as 4–8 blastomeres as well as very late stage embryos as late as hatched blastocysts were being transferred during this period of time.

In addition to the compactness, symmetry, and density that were considered by Shea, Hines, 1976 [16], for embryo evaluation, the later classifications also considered criteria such as the color and shape of blastomeres, signs of degeneration such as vesiculation, the extrusion of blastomeres from the main cell mass, the retardation of embryos, and the integrity of the zona pellucida.

**Table 1:** Evaluation of bovine embryo quality based on morphological characteristics.

Source	Criteria
[16]	“5: excellent appearing 4 3: average appearing 2 1: very poor appearing” rated “*on the basis of compactness, symmetry and density of the blastomeres*”
[17]	“Excellent—judged to be at the normal stage of development at the time of examination; embryos were symmetrical, and blastomeres were polygonal in shape forming a tight mass at the morula stage. Good—similar to excellent embryos but were asymmetrical, contained blastomeres excluded from the main morula mass, or were slightly retarded relative to other embryos recovered from the same donor Fair—embryos were retarded 1 to 2 days in development, had spherical rather than polygonal blastomeres at the morula stage, contained blastomeres of varying sizes, had signs of degeneration such as large vesicles in the cells, and/or were darker or lighter than normal Poor—embryos were retarded 2 or more days in development, had indistinct cell membranes, and/or had more severe faults than the fair embryos”
[18]	“4—embryo is above average in appearance (perfectly symmetrical, even granulation, no deformations in the zona pellucida, no blastomeres extruded) 3—embryo is average in appearance 2—embryo is below average in appearance (uneven blastomere size, extensive blastomere extrusion, evidence of membrane rupture)”
[7]	“Excellent—an ideal embryo, spherical, symmetrical with cells of uniform size, color and texture Good—trivial imperfections such as a few extruded blastomeres, irregular shape, few vesicles Fair—definite but not severe problems, presence of extruded blastomeres, vesiculation, few degenerated cells Poor—severe problems, numerous extruded blastomeres, degenerated cells, cells of varying sizes, large numerous vesicles but a viable-appearing embryo mass”
[20]	“Code 1: Excellent or good Code 2: Fair Code 3: Poor Code 4: Degenerated”

### 2.2. Evaluation for Embryo Staging/Grading

None of the embryo evaluation systems shown in Table 1 recognized the developmental stages of embryos; i.e., an embryo categorized as “excellent” could be a morula, early blastocyst, expanded blastocyst, etc. Even though a coding system that identifies embryo developmental stages had already been published ([21]; Table 2), it was not incorporated into embryo evaluation criteria until the International Embryo Transfer Society (IETS) introduced a two-digit coding system that would uniformly and systematically describe embryos by their stage of development as well as their quality [20] (Supplementary Figures S1–S19). The first digit identified the stage of embryonic development, ranging from 1 (an unfertilized oocyte) to 9 (an expanding hatched blastocyst; Table 2), while the second digit identified embryo quality (based on morphological characteristics), ranging from 1 (excellent/good) to 4 (degenerated; Table 2). For example, an embryo with a 7–1 coding would refer to an expanded blastocyst of excellent/good quality. This two-digit coding system, introduced in 1998, remains the gold standard for embryo evaluation and grading within the bovine embryo production and transfer industry.

## 3. Embryo Evaluation/Analysis—Tools of the Trade

According to the currently accepted norms of bovine embryo evaluation [20], the ideal unhatched bovine embryo should have the following characteristics: the embryo should be compact, spherical, and 150 to 190 µm in diameter (including the 12 to 15 µm thickness of the zona pellucida); blastomeres should be uniform in size, color, and texture; the blastomere cytoplasm should be free of granules and vesicles; perivitelline spaces should be clear and free of cellular debris; and the zona pellucida should be uniform and free of cracks and surface debris (Supplementary Figures S1–S19).

Embryo evaluation is typically performed using a stereomicroscope at 50 to 100× magnification. Embryos are held in a holding dish with holding media and evaluated by rolling it so that the entire embryo and zona pellucida can be evaluated from different angles. However, this type of morphological assessment is considered biased by the subjectivity of the evaluator, i.e., intra- and inter-observer variability [5], and therefore not considered 100% reliable and trustworthy [22,23]. Supporting this notion, differences in ultrastructure [24] and the transcriptome [25] have been demonstrated in morphologically similar, stage- and quality-matched bovine blastocysts. As mentioned earlier, a wide variety of tools have been used to study embryos to understand pre-implantation development and to search for that perfect embryo evaluation method. The following sections address these tools and techniques in detail.

### 3.1. Microscopic Analyses

#### 3.1.1. Light Microscopy

Light microscopy has been one of the most fundamental and widely used tools in biology for nearly two centuries. It is ubiquitously used to analyze both fixed and living cells and is widely regarded as the least invasive method to obtain biological information from living cells [26]. This feature makes it particularly useful, to the point of being absolutely required, for analyzing embryos in research and commercial enterprises that produce bovine embryos for breeding. As stated previously, the standard method for evaluating embryo quality utilizes light microscopy: in typical cases, the direct observation of embryo morphology under a stereomicroscope. However, magnification is limited when using stereomicroscopes, and morphological details can be more readily discerned by observation with compound microscopes utilizing higher magnifications. Indeed, a study conducted in 2002 comparing embryo quality, as evaluated by observation under a stereomicroscope, standard compound microscope, and electron microscope, found that observation under a stereomicroscope leads to the overestimation of embryo quality, as signs of embryonic degeneration are more readily apparent at the higher magnifications available with compound microscopes or the electron microscope [27]. Unfortunately, specimen preparation requirements for observation at higher magnifications under a compound microscope or electron microscope are not compatible with embryo viability.

#### 3.1.2. Differential Interference Contrast (DIC) Microscopy

For most living cells observed under standard light microscopy, the contrast is poor, and they appear almost transparent. It is difficult to discern cellular structures, no matter the ability of the magnifying and resolving power of the lenses in use, unless the cells are fixed and stained with chemical agents that provide contrast. These procedures almost invariably kill living samples. Because of this, microscopists have endeavored to develop optical techniques that provide contrast to cellular structure without requiring the use of fixatives and stains, thus allowing better visualization of living cells. These techniques are commonly used in biological microscopy today and include phase-contrast microscopy, darkfield microscopy, polarized light microscopy, Hoffman modulation contrast, and differential interference contrast (DIC) microscopy.

Developed by Georges Nomarski in the early 1950s [28], DIC utilizes polarized light that is passed through a prism, through the specimen, and then through another prism and depolarized to produce interference that results in one side of the structure that the light has passed through appearing bright and the other side appearing dark. Overall, imaging cells using this technique produces a shadowing effect that gives cells a 3D-relief-like appearance [29].

DIC microscopy is a good option for imaging embryos and live cells. DIC imaging of embryos has been used to create the qualitative scoring systems used today for bovine embryo grading/staging (personal communication, Prof. Matthew Wheeler, UIUC). DIC has been used to study bovine embryos, particularly to study pre-implantation-stage embryo development, to study subcellular components such as lipid droplets and pronuclei, to study the effects on different culture media, and to visualize microinjection procedures (Table 3). As a standalone procedure, DIC use for the analysis of embryos is declining, but its presence continues in combination with other techniques and more complex imaging systems.

#### 3.1.3. Electron Microscopy

The maximum magnification that can be achieved with standard light microscopy is generally no more than 1000×. In contrast, electron microscopy allows magnifications up to around 160,000×. This allows cellular ultrastructure to be clearly visualized. In terms of resolution, while standard light microscopy can resolve structures at about 250 nm in size, the resolving power of electron microscopes provides almost a 1000-fold increase in resolution, getting down to around the 0.2 nm range [40].

The very first study that examined the ultrastructure of pre-implantation bovine embryos was carried out in 1978 [41]. The authors specifically used scanning electron microscopy to study hatching blastocysts that were recovered *unhatched* from cows 7–10 days after estrus and then allowed to hatch during in vitro culture. Many studies have been carried out since then to study various aspects of pre-implantation bovine embryos, including organelle structures in blastomeres [42,43], the nucleologenesis of blastomeres [44,45,46,47], the embryonic cell cycle [48,49], events associated with fertilization (in vitro) and zygote formation [50], the effects of vitrification [51], the effects of different culture media on IVP blastocysts [52,53,54], and the effects of cryopreservation on IVD and IVP blastocysts [55], and to compare IVD and IVP embryos [43,56,57].

A study that compared the accuracy of bovine embryo grading using stereomicroscopy, light microscopy, and transmission electron microscopy (TEM) found that TEM has a better accuracy in identifying “good”- and “fair”-quality blastocysts compared to the other two types [24]. However, this finding has minimal value from a practical application point of view in the dairy industry because fixing embryos for TEM makes them unviable. Therefore, although TEM can be used as a research tool to learn about pre-implantation embryo development, it has little value for routine IVF procedures in the dairy industry [58].

#### 3.1.4. Fluorescence Microscopy

Another modification of light microscopy that is widely used today in biological research is fluorescence microscopy [59]. Fluorescence is the emission of light by a substance that has absorbed light or other electromagnetic radiation. This form of microscopy takes advantage of the ability of different compounds, referred to as fluorophores, to emit light with a monochromatic wavelength when they absorb light with a higher-energy wavelength. By coupling fluorophores with molecular probes that can recognize different proteins or other types of biological molecules, researchers can visualize and quantify the presence of different biological molecules of interest within cells, and they can observe the location and distribution of different molecules within cells, embryos, and tissues in order to gain insights on molecular and cellular functions. As with standard light microscopy, fluorescence microscopy is limited in resolving power to about 200 nm, and techniques for labeling cells with fluorophore-coupled probes are not usually compatible with cell viability. However, there are some techniques available for the fluorescent imaging of living cells and embryos [60].

Fluorescent microscopy has been used to study various aspects of pre-implantation bovine embryo development for simple tasks, such as counting blastomere numbers using DAPI or Hoechst nuclear staining [61,62] or detecting de novo DNA synthesis [63], as well as for more complicated procedures, such as fluorescent in situ hybridization (FISH) for detecting chromosomal abnormalities [63] or the immunodetection of the epigenetic markers H3K9ac and H3K9m2 [62] and the transcription factor POU5F1 [64].

The fluorescent microscopic techniques outlined above produce flat, two-dimensional (2D) images of embryos. In contrast, confocal microscopy and multiphoton laser scanning microscopy (MPLSM) can be used to produce 3D images of embryos, which can be more informative than 2D images. As with standard fluorescence microscopy, confocal microscopy and MPLSM are typically used in conjunction with molecule-specific fluorescence labeling, which allows the identification and study of not only subcellular organelles and components (e.g., lipid droplets and mitochondria) but also the intraembryonic and intracellular localization of proteins/gene products (e.g., transcription factors and lineage-specific markers). Additionally, a relatively new non-fluorescent microscopic technique, Gradient Light Interference Microscopy (GLIM), allows the production of three-dimensional (3D) images. The following sections discuss how these 3D microscopic techniques have been used in the study of pre-implantation bovine embryos.

#### 3.1.5. Confocal Microscopy

The widespread use of fluorescent confocal microscopy on cells and tissues started in the late 1980s [65,66] because of its great applicability to quantitative analysis [67]. In conventional fluorescence microscopy, the entire sample is exposed to the excitation light source, and the entire sample emits fluorescence. When observed under the microscope and images are acquired, some out-of-focus fluorescent signals interfere with the in-focus light. Additionally, the exposure of whole live cells and embryos to this light source can cause phototoxicity, resulting in decreased cell viability. Through the use of optical sectioning technology, the confocal microscope allows the exposure and gathering of the fluorescent signal from a relatively narrow focal plane of the sample. By gathering images of focal planes throughout the sample (commonly referred to as z-stacks), three-dimensional images can be created. This can be applied to either fixed or live embryos [67,68]. There is still some risk of phototoxicity due to the power of the excitation source and the length of the scanning time, but it is possible to minimize this challenge by properly selecting the microscope, settings, and sample processing method [68]. Additionally, bovine embryos are able to maintain viability by successfully overcoming certain levels of phototoxicity [69]. Of the different confocal microscopic optical sectioning techniques available, the spinning-disk confocal system is considered the preferred technique for live imaging [68,70].

Confocal laser scanning has been widely applied to fixed and live samples to visualize specific cellular structures using fluorescent labeling or a label-free approach [68]. In pre-implantation bovine embryos, confocal microscopy has been used for a variety of applications, as summarized in Table 4.

#### 3.1.6. Multiphoton Laser Scanning Microscopy (MPLSM)

Even though confocal microscopy has been used successfully for imaging the subcellular components, transcription factors, lineage-specific markers, and metabolites of bovine embryos, it can still be phototoxic to live cells/embryos and potentially compromise their viability. In contrast, MPLSM can be used for imaging fluorescent markers in relatively thick specimens, such as mammalian embryos, without compromising their viability [84]. Further, MPLSM is considered superior to confocal microscopy for scanning thick specimens, such as mammalian embryos, that exhibit significant light-scattering properties. These beneficial characteristics of MPLSM are primarily due to multiphoton systems using infrared lasers as their excitation source. Infrared light is lower in energy and scatters much less compared to light with excitation wavelengths used in confocal or standard fluorescence microscopy [85]. MPLSM has been used to image cellular organelles such as mitochondria in bovine blastomeres [85]; however, its use for studying pre-implantation bovine embryos has been very limited, possibly due to the high expenditure on equipment and the need for technical expertise [69].

Our group has begun preliminary studies using simultaneous label-free autofluorescence-multiharmonic (SLAM) microscopy [86], which is a single-excitation source nonlinear imaging platform to analyze the metabolic pathways in bovine embryos (Tu and Wheeler, 2023, personal communication).

#### 3.1.7. Gradient Light Interference Microscopy (GLIM)

There are two major deficiencies in many of the microscopic techniques discussed earlier. First, the imaging of optically dense specimens such as embryos with conventional microscopy results in multiple scattering that scrambles the optical field, giving rise to low-contrast micrographs that often lack cellular-level detail [11,87]. Secondly, microscopic techniques such as TEM and fluorescent microscopy cannot be used to image live embryos.

GLIM can be used to overcome both of the above deficiencies. On the one hand, it can be used for the real-time imaging of *thick* embryos, overcoming multiple scattering. On the other hand, as a label-free method, GLIM can be used to image embryos without affecting their viability, thus enabling the technology to be used in the evaluation of in vitro produced bovine embryos prior to freezing and/or transfer [88]. Additionally, GLIM is a relatively rapid technique in that it takes ~1 min for image acquisition and >5 min for the virtual reconstruction of the image. GLIM can also be used to track and follow lipid droplet movement, which could be a possible marker of embryo quality [29], as lipid movement has been linked to protein and lipid exchange between cellular compartments in embryos of certain other species [89].

GLIM is a type of quantitative phase imaging (QPI) that combines DIC microscopy with low-coherence interferometry and holography. It is considered superior to other QPI methods, such as Spatial Light Interference Microscopy (SLIM), for imaging optically thick samples such as embryos. GLIM can be used to obtain high-contrast three-dimensional, white-light, tomographic imaging of both thin samples, such as single cells, and relatively thick specimens, such as bovine embryos. GLIM has been used to obtain 3D stacks of bovine embryos at different development stages over several days using time-resolved tomography. Using GLIM tomography, researchers were able to study individual blastomeres, their plasma membranes, gaps between plasma membranes of different blastomeres, lipid droplets in blastomeres, etc. [58]. Considering these favorable properties, GLIM has the potential to become a valuable tool for the evaluation of in vitro produced bovine embryos.

#### 3.1.8. Time-Lapse Monitoring (TLM)

The previously described microscopic techniques allow the spatial evaluation of embryos. However, embryo development is a dynamic process, and critical stages of development may go unnoticed with such traditional, one-time morphologic assessments [90]. In contrast to these one-time morphological evaluations, time-lapse monitoring (TLM) allows embryos to be evaluated temporally as well. TLM facilitates the study of morphokinetics, allowing the correlation of early events with subsequent development and even pregnancy outcomes. Embryos could be inspected multiple times with conventional microscopy too; however, such frequent evaluations can be detrimental to embryos owing to the frequent handling and exposure to changes in temperature and gas concentrations. In contrast, TLM allows the frequent and non-invasive imaging of developing embryos while maintaining *incubator conditions* [91].

The first reports on the TLM of bovine embryos were published in the early 1980s [92,93]. Morulae collected from uterine horns were allowed to grow and hatch in vitro while being continuously monitored. These authors observed that pulsatile expansion and contraction was *not a necessary condition* for the hatching of the bovine blastocyst, an observation that could not have been made with conventional *one-time microscopy*. Since then, TLM has been used extensively to study pre-implantation bovine embryo morphokinetics, as summarized in Table 5. Some of these studies observed that morphokinetic indicators (MKIs), such as the timing of the first cleavage, the number of blastomeres at the first cleavage, and the number of blastomeres at the onset of the lag phase, could be used as markers for predicting blastocyst quality and the pregnancy outcome [94,95]. Some have even proposed MKIs as a superior substitute to the IETS’ morphology-based grading system [81].

TLM studies from the 1980s–2000s mostly used a cinematographic chamber placed on an inverted microscope. However, with the advancements in technology, high-resolution imaging equipment has been integrated into fully functional incubators, enabling embryo-safe recording, i.e., TLM (e.g., Primo Vision, Vitrolife, Goteborg, Sweden, and Real-Time Cultured Cell Monitoring System with Multiple-Point Imaging Capture (CCM-MULTI), Astec, Fukuoka, Japan). These systems have been successfully used to study pre-implantation bovine embryo development. Taking TLM a step further, several recent studies have used confocal laser microscopy for time-lapse live-cell imaging [81] to shed light on aspects of bovine embryo development in both temporal and spatial dimensions. The advantages of four-dimensional (4D) imaging over other microscopic techniques are numerous and have been reviewed elsewhere [96].

**Table 5:** Applications of time-lapse monitoring in bovine embryo evaluation and analysis.

Applications of TLM in Bovine Embryo Analysis	Source
To compare cleavage intervals of healthy embryos and degenerate embryos	[97]
To study development kinetics of embryos derived from calf oocytes	[98]
To study the effects of activin A and follistatin on developmental kinetics	[99]
To study effects of glucose on developmental kinetics of male and female embryos	[100]
To compare developmental kinetics of IVD and IVP embryos	[101]
To study dynamics of the 4th cell cycle coincident with EGA	[102]
To compare kinetics of initial cleavage patterns in viable and non-viable embryos	[103]
To study embryo development in well-of-the-well (WOW) systems	[104]
To identify morphokinetics predictive of blastocyst quality and pregnancy outcome	[94]
To study effects of abnormal cleavage patterns on morphokinetics and growth potential	[105]
To study development kinetics of IVP embryos fertilized with sex-sorted semen	[106]
To study pronuclear morphokinetics	[107]
To study effects of first zygotic cleavage dysmorphisms on the metabolomic profile	[108]
To correlate embryo morphokinetics with their transcriptomic profiles	[109]
To study morphokinetics of embryos derived from vitrified bovine oocytes	[90]

In summary, every microscopic technique that has been used to study bovine embryos to date has its unique pros and cons. Conventional light microscopic methods are simple to use, relatively inexpensive, and non-invasive and therefore can be used without affecting embryo viability, even in locations with limited infrastructural facilities. However, morphological evaluation by conventional light microscopy may be limited by its magnification, resolution, and contrast. On the other hand, technologically advanced microscopic methods such as TEM and fluorescent microscopy are not compatible with maintaining the viability of embryos. Therefore, even though these techniques can be used to study embryo physiology, even at the molecular level, they are invasive methods that have no real-world applications in evaluating live embryos prior to freezing or transfer. GLIM can overcome many of the limitations discussed above for other microscopic techniques. However, it involves the use of highly sophisticated and expensive equipment, as this technology is still relatively new. Similarly, the imaging equipment used in TLM is also sophisticated and expensive. Therefore, it is unlikely that these techniques will be adopted by the dairy and beef industries at the national and global scales at the current time.

### 3.2. Non-Microscopic Analyses

#### 3.2.1. Pre-Implantation Genetic Diagnosis (PGD)

The term Pre-implantation Genetic Diagnosis (PGD) was coined in the late 1980s [110] to describe a procedure where a biopsy of embryonic cells is tested for *diagnosing* a genetic trait. However, similar procedures took place as early as 1968 when 5–6-day-old pre-implantation-stage rabbit blastocysts were sexed by the microscopic identification of *sex chromatin* in biopsied trophoblast cells [111]. In fact, until the early 1990s, most genetic *diagnoses* of pre-implantation embryos were aimed at sex determination because transferring a known female embryo would lead to numerous benefits to livestock industries [112]. This was especially important in the *pre-sexed semen era*, where embryo sexing was the only viable option if IVD or IVP embryos were to be sexed before being used in various livestock embryo transfer programs.

Initial studies on embryo sexing of pre-implantation bovine embryos involved in situ hybridization with a Y-chromosome-specific DNA probe [113,114]. However, with the advent of the polymerase chain reaction (PCR; [115]), it became the method of choice for sex determination and other pre-implantation genetic analyses. The first PCR-based sexing of a pre-implantation bovine embryo took place in the early 1990s using DNA extracted from a single biopsied blastomere from a 16–32-cell morula [116]. Similar studies followed, with blastomere biopsies from 8- to 16-cell [117] and 16- to 32-cell bovine morulae [118] and trophoblast cell biopsies from bovine blastocysts [119]. The first bovine-embryo-related study to utilize PCR for a non-sexing purpose studied the temporal expression dynamics of the gap junction gene *Cx43* [120]. Since then, PCR (especially quantitative real-time PCR) has been used extensively to study various aspects of pre-implantation bovine embryos, including EGA [80,121], early lineage specification [80,122], and the effects of superovulation on embryos [123], and to validate gene expression data from dozens, if not hundreds, of microarray and RNA-seq studies.

An important prerequisite for successful PGD is the need to perform embryo biopsy without affecting embryo viability/competence. As reviewed elsewhere, pregnancy rates of 31–63% have been obtained by different groups after transferring biopsied pre-implantation bovine embryos [124]. Only a handful of studies have directly compared pregnancy rates resulting from transferring biopsied and intact embryos. One of them observed a ~15% reduction in the pregnancy rate when transferring biopsied blastocysts, while others did not (Table 6). This lack of agreement between studies could be attributed to varying conditions between the studies, such as the number of biopsied blastomeres, the gestation length at the time of pregnancy testing, and variations among recipients.

#### 3.2.2. Omics-Based Embryo Analyses

PGD is carried out to *diagnose* a specific trait of interest (e.g., sex, chromosomal aberration, or a genetic disease, esp. in humans). In contrast, with the advancements in the “omics”, pre-implantation embryos can be screened for entire genomes (also known as pre-implantation genomic/genetic screening: PGS), transcriptomes, epigenomes, proteomes, and metabolomes. These tools can provide quantitative and statistically accurate insights about not just a handful of molecules but hundreds, if not thousands, of potential biomarkers. This massive amount of data can also be used to understand functional relationships among identified *markers*, i.e., in cellular pathways/networks [128,129]. As such, omics-related studies have been carried out extensively over the last three decades in search of that gold standard test for the pre-transfer evaluation of embryos and in an attempt to further understand pre-implantation embryo development. The following sections examine the various types of studies that have taken place in the “omics” related to pre-implantation bovine embryos.

##### Genomic Studies

Genomic selection is widely used in dairy [130] and beef [131] breeding programs today. Genomic estimated breeding values (GEBVs) are currently calculated after the birth of calves [132]. This practice creates some degree of *wastage* in the form of calves that do not meet the expected minimum genetic merit/GEBV and all the resources that have gone into bringing such calves to life, e.g., the dams/surrogates that gestated them. These wastages can be minimized by using pre-implantation genomic screening (PGS) to screen blastocysts for favorable health, production, and reproduction traits prior to their transfer to a recipient [125,133,134]. Such an approach will enable the transfer of only the best embryos, not only avoiding the previously mentioned wastages but also improving the selection intensity and shortening the generation interval, leading to faster genetic gains [124,125,132]. Large-scale commercial applications of PGS are possible today due to the availability of affordable single-nucleotide polymorphism (SNP) chips [124,132,135] and GEBVs with reliabilities approaching 70% [125].

Genomic studies using SNP genotyping have been carried out in pre-implantation bovine embryos mainly to test for aneuploidy [132,135,136,137], chromosomal aberrations [136,138], and karyomapping [135,139] to date. After identifying euploid embryos for transfer (from karyomapped IVP embryos), live calves were born, demonstrating that genomic/SNP studies indeed have ground-level applications [135].

##### Microarray for Transcriptome Profiling

Genetic evaluations of pre-implantation embryos that started in the form of PGD evolved to high-throughput methods that could rapidly screen entire transcriptomes around the dawn of the 21st century. These transcriptome-wide studies not only allow the study of many thousands of genes simultaneously but also help make biological sense of the underlying pathophysiology by identifying biological pathways or networks that are differentially affected between groups of samples [129].

The very first transcriptome-wide studies involving bovine embryos were carried out using suppression-subtractive hybridization (SSH) in 2002–2004 [140,141]. The first microarray study on bovine embryos was reported in 2004 [142]. The research group compared the transcriptomes of IVD and IVP blastocysts using a custom-made bovine microarray. The next decade saw several dozen microarray studies carried out to study various aspects of pre-implantation bovine embryo development using several commercial and *homemade* cDNA and oligonucleotide microarray platforms (Table 7).

In some of these studies, RNA was extracted from whole embryos [142,143,144], making it a useless approach for pre-transfer embryo evaluation. Nevertheless, such studies are still useful for further studying pre-implantation embryo development from a research perspective. However, many other microarray studies have been carried out by extracting RNA from biopsies of ~30–40% of the intact embryo. During one such study, the remaining 60–70% of the embryos were allowed to expand in vitro, and the resulting demi-blastocysts were transferred to recipients. A 30% pregnancy rate was achieved in this study. Further, by comparing the transcriptomes of blastocysts that succeeded in a live birth *vs*. those that did not, they found potential candidate genes that are associated with the post-transfer pregnancy outcome [145,146]. Such gene expression signatures may serve as markers for evaluating pre-transfer embryos. Therefore, microarray technology indeed has the potential to be used in the pre-transfer evaluation of embryos in the dairy and beef industries.

**Figure 3:**
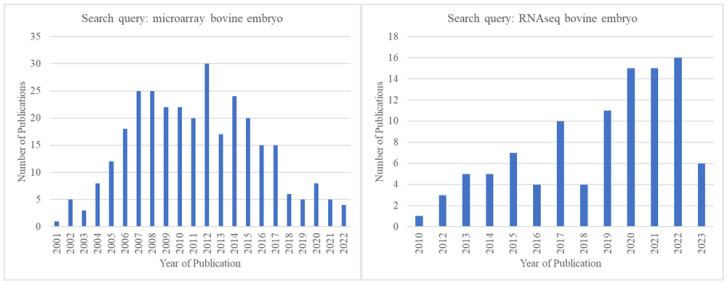
Comparison of the number of publications identified in Pubmed.gov (https://pubmed.ncbi.nlm.nih.gov/, accessed on 15 May 2023) by year for the search queries “microarray bovine embryo” and “RNA-seq bovine embryo” showing that after the year 2012, microarray-related publications declined, and RNA-seq-related publications increased.

With the advent of RNA-sequencing (RNA-seq) technology, a transcription profiling approach superior to microarrays in many ways [176,177], the popularity of microarrays gradually started decreasing. There seemed to be an obvious shift to RNA-seq from microarrays starting in ~2012, even among those engaged in pre-implantation bovine embryo research. This is clearly reflected in the publication record on Pubmed.gov (Figure 3).

##### RNA Sequencing (RNA-Seq) for Transcriptome Profiling

The very first RNA-seq study of bovine embryos was carried out in 2010 to compare the transcriptomes of *healthy-looking* and *degenerated* IVP blastocysts using the Genome Analyzer platform by Illumina [178]. Since then, several dozen RNA-seq studies have been carried out to study various aspects of pre-implantation bovine embryo development using several commercially available platforms (Table 8). Some of these studies used pooled RNA [25,178,179,180], some used RNA from individual embryos [181,182], and others used RNA from embryo biopsies [183,184], including from those as little as single blastomeres [185,186]. However, extracting minute quantities of RNA from single cells will require the RNA to be amplified, which can lead to undesirable complications.

Transferring 12 demi-embryos remaining after biopsy for RNA extraction, Zolini, Block, 2020 [183], achieved 5 pregnancies (41.7%) at 60 days of gestation, demonstrating that blastocysts tested by RNA-seq can indeed be used for the pre-transfer evaluation of bovine embryos.

##### Proteomics

The embryonic proteome is generally considered a better reflection of the actual embryo dynamics than the transcriptome for two reasons. First, it is proteins, metabolites, and other small molecules that are usually the final players in cellular/physiological events. In contrast, transcripts are usually intermediate players. Second, embryos are transcriptionally quiescent during the first few cleavages until EGA takes place. During this phase, embryo development is mainly controlled by oocyte-derived RNAs and proteins [203,204]. In contrast to the relatively invasive methods of embryo evaluation discussed earlier (e.g., genomics, transcriptomics, and even certain types of microscopy), proteomic studies can be carried out relatively non-invasively by analyzing embryos’ secretomes present in the culture media.

Proteomic analyses of pre-implantation embryos have been carried out to study early lineage specification [204], EGA [203], stage-specific proteome dynamics during pre-implantation development [203,205], differences between IVP and IVD embryos [206], proteomes of the blastocoel fluid [207], primitive yolk sac fluid [208], and trophectoderm [209], and to identify embryotrophic factors in the secretome that can serve as biomarkers of high-quality embryos [210].

Traditionally, proteomic profiling was performed using 2D gel electrophoresis and Western blotting. However, modern studies use a variety of mass-spectrometry-based technologies, such as Matrix-Assisted Laser Desorption Ionization–Time-Of-Flight Mass Spectrometry (MALDI-TOF MS), Liquid Chromatography with Tandem Mass Spectrometry (LC-MS-MS), Nanoliquid Chromatography Coupled with Tandem Mass Spectrometry (nanoLC-MS/MS), and Nano-HPLC Tandem Mass Spectrometry.

##### Metabolic Profiling

The metabolome is a downstream representation of the transcriptome and proteome because metabolites are the final products of cellular functions mediated by the transcriptome and proteome. As such, a strong association has been observed between embryo physiology and its metabolic profile, prompting researchers to develop metabolomics-based biomarkers. Similar to proteomics, the metabolic profile of pre-implantation embryos can be analyzed in a highly non-invasive fashion, making it a powerful tool for pre-transfer or pre-cryopreservation embryo evaluation. The metabolic profile for a specific metabolite can be measured as the quantity (i) consumed/taken up or (ii) released by the embryo. The evaluation of the embryo microenvironment, i.e., spent culture media, can give a reasonably accurate picture of both of the above measurements for individual metabolites or the entire metabolome [10,23,211].

The very first report on measuring metabolites in bovine blastocysts goes back to 1988 [212] when a team of researchers measured glucose, pyruvate, and glutamine, substrates related to energy metabolism. Initial studies in the 1980s and the first half of the 1990s focused on substrates related to energy metabolism, such as glucose, pyruvate, and glutamine. Later on, researchers studied oxygen, too, as an indicator of overall metabolic activity because the generation of cellular energy *via* mitochondrial oxidative phosphorylation is oxygen-dependent [213,214]. Over the last decade, many other candidates have been identified and tested by various research groups (Table 9).

During the pre-2000 era, mostly fluorometric or reverse-phase HPLC assays were used to quantify individual metabolites, such as glucose, lactate, pyruvate [215], and amino acids [216]. In addition, tools such as nanorespirometers (to measure oxygen consumption; [217]), embryoscopes with built-in oxygen microsensors (to measure oxygen consumption; [218]), REDOX sensors (to measure the production of ROS; [218]), and multi-sensor systems that can measure oxygen, glucose, and lactate concentrations simultaneously [219] had also been developed/used to evaluate the metabolic profiles of bovine embryos.

With massive developments in biomedical technologies, these techniques were replaced by mass spectrometry (MS)-based metabolomics, which allows the mass detection of metabolites, i.e., the metabolome, concurrently (e.g., >10,000 metabolites; [220]). These processes typically consist of two steps: separation using chromatography, followed by detection using MS. Examples of such methodologies used to date are Ultra-High-Performance Liquid Chromatography–Time-of-Flight Mass Spectrometry (UHPLC-TOF MS; UHPLC-TOF MS; [220,221]), Gas Chromatography Quadrupole Time-of-Flight Mass Spectrometry (GC-qTOF; [12]), Fourier Transform Infrared Spectroscopy (FTIR; [222]), and Raman Spectroscopy [223]. In addition to MS-based techniques, high-resolution nuclear magnetic resonance (^1^H NMR) spectroscopy [224,225] has also been used for metabolomics studies of pre-implantation bovine embryos.

Metabolomics has been used not only to identify embryonic viability markers of bovine embryos but also to determine the sex of the embryo [220,221,222,224,226].

**Table 9:** Metabolites analyzed in bovine embryos.

Analyzed Metabolite or Indicator of Metabolism	Source/s
Glucose	[212,215,227,228,229,230]
Pyruvate	[212,215,225,228,229,231]
Lactate	[215,224,225,229,231]
Amino acids	[212,216,220,221,224,225,226,227,228,229,231,232,233]
Fatty acids	[221,234,235]
Oxygen	[217,218,229,236]
Reactive oxygen species (ROS)	[218]
Myo-inositol	[224,231]
Citrate	[224,231,233]
Formate	[224,231]
Prostaglandins	[221]
Biotin	[220]

#### 3.2.3. Nuclear Magnetic Resonance (NMR)

Nuclear magnetic resonance (NMR) is used extensively in hospitals and research centers for non-invasive in vivo diagnostic procedures (e.g., commonly known as MRI scanners). In contrast to the ionizing radiation of X-ray and computed tomography (CT scanning), which can be harmful to live cells, the non-ionizing radiation of NMR is considered harmless or minimally harmful to live cells and therefore can be used to identify and quantify biologically important markers in intact cells, embryos, extracts, or the secretome [237,238]. In fact, several research groups have already used NMR spectroscopy to discover metabolome-based biomarkers that can predict fetal sex and the future development of pre-implantation bovine embryos [224,225,231,238]. Once these biomarkers are identified by NMR, simple enzymatic assays can be developed to identify/quantify such markers without the need for sophisticated NMR equipment [237]. As such, there is great potential for metabolomic biomarkers, identified by NMR or other means, to have practical applications in non-invasive embryo evaluation in the bovine ET industry.

ART-related applications of NMR, including those used for the evaluation of embryos, have been reviewed in detail elsewhere [237].

#### 3.2.4. Less Commonly Used Tools

In addition to the methods described above, pre-implantation bovine embryos have been studied using a variety of other tools too (Table 10).

## 4. Discussion

Nearly 1.5 million bovine embryos were transferred in 2021. Of these transfers, nearly 80% (~1.2 million) involved IVP embryos [2]. Despite the massive advancements in every aspect of IVP, only ~27% of them can be expected to produce a live calf [4]. While the causes of pregnancy failures are multi-factorial, part of the problem is the inadvertent transfer of low-quality embryos due to our incompetence in accurately distinguishing high-quality embryos from low-quality ones. The current method of choice for embryo evaluation, i.e., morphological evaluation using a stereomicroscope, is considered to be subjective and to have poor accuracy and reproducibility [1,5]. Over two decades of research has gone into discovering a replacement tool for manual morphological evaluation, yet nothing concrete has come out that is adoptable at the ground level.

### 4.1. Microscopic Techniques

Of the visualization methodologies discussed, stereomicroscopy is the most commonly used tool for the pre-transfer/pre-cryopreservation evaluation of bovine embryo/blastocyst quality. However, the methodology is plagued by human subjectivity and poor accuracy. Electron microscopy and fluorescent microscopy have contributed immensely to the current knowledge about pre-implantation bovine embryo ultrastructure and the intraembryonic/intracellular localization of mRNAs and proteins (through FISH, immunocytochemistry, etc.). However, because sample preparation procedures make the embryos non-viable, they are both invasive techniques of no value for live embryo evaluation.

GLIM is superior to the abovementioned microscopic techniques because it is label-free and non-invasive and captures real-time 3D images of thick embryos, overcoming multiple scattering. Additionally, it takes ~1 min for image acquisition and <5 min for the virtual reconstruction of the image. As such, GLIM can be used for live embryo evaluation without affecting their viability. However, the equipment is expensive and sophisticated and requires a high level of technical expertise to operate. Therefore, it is highly unlikely to have practical applications in the dairy or beef cattle industry in the immediate future.

TLM allows the temporospatial evaluation of embryos and thus can be used to study embryo morphokinetics. Several MKIs predictive of embryo viability/quality have been discovered using TLM; however, the procedure requires embryos to be cultured and tracked individually using expensive imaging equipment. Therefore, however powerful a tool it may be, TLM too is highly unlikely to be adopted by the masses in the immediate future.

### 4.2. Non-Microscopic Techniques

The most common omics-based methodologies that have been used to study pre-implantation bovine embryo development to date are related to transcriptomics and metabolomics. Relatively few studies have been carried out using SNPs, proteomics, and epigenomics. There is no doubt that many of the discussed “omics” technologies have succeeded in discovering embryo viability/quality markers. However, when it comes to in-field applications of these technologies, they pose more questions than they provide answers. For example, if transcriptomics were to be adopted for in-field embryo evaluations, would it be realistic to biopsy and extract RNA from 2 million embryos (the approximate annual cattle embryo production) and then to run 2 million microarrays/RNA-seq? Would these steps take place at the embryo production/collection facilities? Or would embryos get transported to a different facility? If they are transported out, embryos may need to be frozen for transporting and thawed out before testing. Because there will be an interval of several days between the biopsy and the availability of results after bioinformatic analyses, embryos will need to be refrozen, and the ones that *pass the tests* will need to be re-thawed before ET. It is well known that freezing and thawing, let alone refreezing and re-thawing, can have undesirable effects on embryo quality/viability [263]. Compared to transcriptomics testing, proteomics and metabolomics are considered non-invasive because it is the culture medium that is analyzed and not the embryo *per se*. However, these require embryos to be cultured individually for their individual secretomes to be analyzed. If proteomics or metabolomics were to be adopted at the ground level, would it be practically possible to culture two million embryos individually using WOW systems? Additionally, even if this mammoth feat could be accomplished, the same set of challenges as with transcriptomics will need to be dealt with.

Therefore, it is clear that while these tools are extremely powerful and useful for understanding pre-implantation embryo development/physiology, it is almost impossible for them to have direct in-field applications in live embryo evaluation.

### 4.3. The Ideal Embryo Evaluation Tool

One of the key characteristics of the ideal embryo evaluation tool would be its adoptability by the thousands of embryo-producing/collecting facilities. Such a tool should fulfill most, if not all, of the following seven criteria:

1. The method should provide accurate results;
2. It should be non-invasive;
3. It should be objective (minimize human subjectivity);
4. It should be low-cost (initial and ongoing costs);
5. It should be technically simple enough to be carried out at the embryo production facility itself;
6. The evaluation should be completed by blastulation so that high-quality blastocysts can be immediately identified for direct transfer, without a lag period like in the case of omics-based techniques;
7. The results should be available within hours of sample collection so that a second freeze–thaw cycle can be avoided.

Based on Table 11, which compares the common tools of embryo evaluation according to the above criteria, none of the tools fulfill all criteria. However, TLM and AI-based microscopy satisfy the highest number of favorable criteria (green boxes).

TLM fulfills a criterion that others do not: i.e., TLM can complete the embryo evaluation by the time that blastulation takes place by virtue of analyzing the morphokinetics of the first few cleavages. TLM studies have been used to demonstrate that MKIs such as the timing of the first cleavage, the number of blastomeres at the first cleavage, and the number of blastomeres at the onset of the lag phase have a strong correlation with post-transfer pregnancy rates [94,95]. These pre-blastulation observations have even led some to argue that MKIs can replace the IETS’ morphology-based grading system [81]. However, the major obstacle to using MKIs is the need to keep track of individual embryos during culture using WOW systems and sophisticated and expensive TLM imaging equipment. Therefore, we emphasize the need for future research to focus on designing affordable imaging alternatives that can be incorporated into existing incubators. A standalone camera or a smartphone that can be placed inside the incubator and is capable of communicating with a computer via WiFi may suffice for this purpose. For example, de Souza Ciniciato, Takahashi, 2017 [255] and Guilherme, Pronunciate, 2018 [252], used a smartphone to capture digital images of blastocysts with limited success. Considering that a successful embryo evaluation method will need to be adopted by the masses and that everybody has access to smartphones nowadays, we believe that further research needs to be carried out to understand how image capture using smartphones can be used in embryo evaluation, especially in TLM. Smartphone-compatible macro lenses claiming up to 400× magnification and timer-based photography applications are available on the market that may suffice for this type of relatively simple application. Smartphone-based biomedical applications are currently being used in various diagnostic, histopathological, and microbiological applications [264,265,266].

AI-based automated microscopy has been used to process and analyze images in an objective and automated fashion. Using artificial neural networks (ANNs), genetic algorithms (GAs), and automated image processing, embryo quality was classified with a high success rate [1,253]. We believe that this technology has a high potential for *in-field* embryo evaluation considering its benefits outlined in Table 11. It is well established that subtle variabilities in color, brightness, shape, and features escape the human eye [267] simply due to limitations in the physiological capacity of the human eye and variabilities in the embryologist’s accuracy, experience, and mood [7]. Automated image processing with objective image analysis, aided by machine learning, can overcome these limitations of the human eye.

Certain commercial TLM systems already make use of automatized image processing to a certain degree. We believe that if AI-based evaluation can be combined with TLM in a manner affordable to the masses, it has the potential to be used for *in-field* embryo evaluation.

### 4.4. Application of Omics-Based Tools in Field Conditions

In contrast to AI-based microscopy and TLM, omics-based technologies have major limitations that prohibit the direction application of these tools under field conditions, e.g., the need for technical expertise for operation and the need for additional freeze–thaw cycles. However, this does not mean that omics-based methods, GLIM, or NMR cannot be used for in-field embryo evaluation. Even though they cannot be used directly *in-field*, they can be used indirectly to narrow down embryo viability biomarkers, which may be detected *in-field* using simpler methodologies. For example, using mass-spectrometry-based proteomics screening of culture media, Raes, Wydooghe, 2023 [210], found that media conditioned by excellent- and good-quality bovine embryos, but not poor-quality embryos, contain cathepsin-L. If cathepsin-L is to be adopted as a bovine embryo viability/quality biomarker for embryo evaluation, mass spectrometry need not be carried out at the ground level. Instead, a simple protein detection methodology such as ELISA, which is non-invasive, simple, affordable, and adoptable by the masses, could be developed for the in-field detection of cathepsin-L. Alternatively, culture media could be developed that change color in the presence of high concentrations of cathepsin-L.

The majority of studies carried out to date have limited their objectives to finding an association between biomarker signatures and blastocyst quality. Only a handful of studies have gone on to correlate biomarker signatures with pregnancy outcomes. We strongly recommend that future studies emphasize investigating correlations between pre-implantation embryo biomarker signatures and pregnancy outcomes. This applies not only to omics-based biomarkers but also to other tools, including microscopic techniques.

## 5. Conclusions

**Figure 4:**
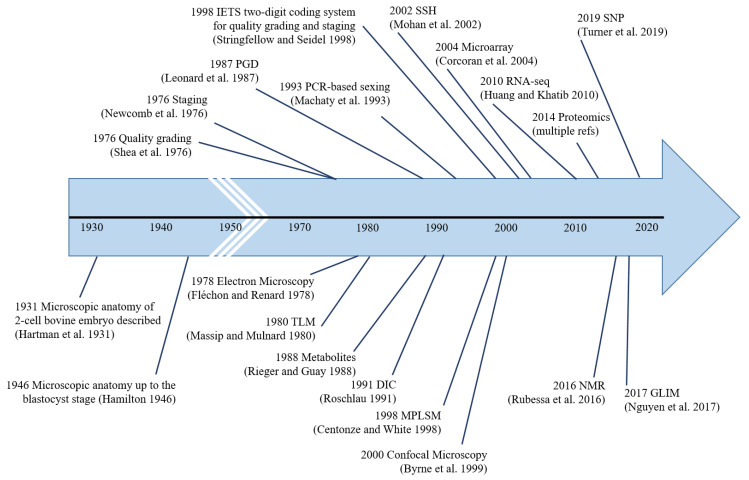
Major milestones in pre-implantation bovine embryo analysis. The first instance of the use of each methodology is indicated, i.e., microscopic anatomy [13,14], quality grading [16], staging [21], electron microscopy [41], time-lapse microscopy [92], genetic-testing [113], metabolite-testing [212], differential interference contrast microscopy [30], PCR-based sexing [116], IETS two-digit coding system [20], multi-photon laser scanning microscopy [85], confocal microscopy [71], suppression subtractive hybridization [140], microarray [142], RNA-seq [178], proteomics, nuclear magnetic resonance [231], gradient light interference microscopy [58], and single nucleotide polymorphisms [139].

Pre-implantation bovine embryo evaluation has come a long way, from simple microscopic analysis in 1931 to PCR-based analyses in the 1990s to omics-based analyses in the 21st century (Figure 4). The ideal embryo evaluation tool should be not only accurate, objective, non-invasive, and affordable but also simple enough to be adopted by the thousands of embryo production/collection facilities worldwide. As such, under the current circumstances, it is highly unlikely that transcriptomics, proteomics, metabolomics, GLIM, NMR, etc., can be directly used for *in-field* embryo evaluation. However, biomarkers identified by these methodologies could be used for *in-field* embryo evaluation by devising simple, affordable *benchtop* techniques. Further research on TLM and AI-based automated image processing is warranted to make them more affordable (esp. TLM), as we see great potential for these methodologies to be adopted by the masses, provided they can be made affordable.

## Figures and Tables

**Table 2:** Coding based on developmental stages of bovine embryos.

Source	Code	Criterion/Definition
[21]	1	2-cell embryo
	2	4-cell embryo
	3	8-cell embryo
	4	Morula (16–32 cells)
	5	Morula (32–64 cells)
	6	Blastocyst
	7	Hatched blastocyst
[20]	1	Unfertilized
	2	2- to 16-cell embryo
	3	Early morula
	4	Morula
	5	Early blastocyst
	6	Blastocyst
	7	Expanded blastocyst
	8	Hatching blastocyst
	9	Hatched blastocyst

**Table 3:** Applications of DIC microscopy in bovine embryo evaluation and analysis.

Applications of DIC in Bovine Embryo Analysis	Source
Visualization of pronuclei	[30]
Evaluation of embryos between days 4 and 8 of culture	[31]
Evaluation of cleavage	[32]
Visualization of lipid droplets	[33]
Comparison of 8-cell-stage embryos in different culture media and visualization of cytoplasmic lipid droplets	[34]
Visualization of DNA microinjection into the male pronucleus	[35,36]
Observation of the development of individual embryos	[37]
Observation of cytoplasmic lipid droplets	[38,39]

**Table 4:** Applications of confocal microscopy in bovine embryo evaluation and analysis.

Applications of Confocal Microscopy in Bovine Embryo Analysis	Source
*Using targeted fluorescent labeling*	
TUNEL labeling of apoptotic nuclei	[37,71,72]
Intracellular localization of nucleolar proteins	[44]
Characterization of the zona pellucida	[73]
Intracellular localization of Nalp5/Mater	[74]
Study of DNA methylation patterns	[75]
Intracellular localization of IGF1R, IGF2, and IGF2R	[76]
Intracellular localization of pluripotency and lineage-specific markers NANOG, OCT4, CDX2, and GATA6	[77,78,79,80]
Non-invasive long-term live-cell imaging of IVP embryos	[81]
Characterization of mitochondrial activity and distribution of lipid droplets	[82]
*Using label-free imaging*	
Detection of autofluorescence of FAD and NAD(P)H (to assess metabolic status)	[83]

**Table 6:** Comparison of pregnancy rates resulting from transfer of biopsied and non-biopsied bovine embryos.

Source	Type of Embryo	Number of Embryos	Pregnancy Rate (PR)	Observations
Mullaart and Wells, 2018 [125]	Biopsied (IVD)	1190	46%	PR different at 5 months (*p* < 0.05)
	Intact (IVD)	13,067	54%	
de Sousa, da Silva Cardoso, 2017 [126]	Biopsied (IVD)	380	54%	PR not different at 60 days (*p* > 0.05)
	Intact (IVD)	229	56%	
	Biopsied (IVP)	91	26%	PR not different at 60 days (*p* > 0.05)
	Intact (IVP)	227	20%	
Fisher, Hyndman, 2012 [127]	Biopsied (IVP)	42	43%	PR not different at 65 days (*p* > 0.05)
	Intact (IVP)	42	38%	

**Table 7:** Applications of microarray technology in bovine embryo evaluation and analysis.

Microarray Platform Used	Objective/s of the Study	Source
Bovine cDNA microarray developed in-house	To compare IVD and IVP blastocysts	[142,143]
BlueChip cDNA microarray, Universite Laval, Quebec, Canada	To compare in vivo derived 2-cell embryos, 8-cell embryos, blastocysts, and GV oocytes	[144]
	To discover genes that will predict the post-transfer fate of embryos	[145,146]
	To study the effects of embryo microenvironment using a well-of-the-well (WOW) system	[147]
	To compare IVD and IVP pre-implantation stages (2-, 4-, and 8-cell stages, morulae, and blastocysts)	[148]
	To compare blastocysts derived from IVP and parthenogenesis	[149]
Affymetrix GeneChip^®^ Bovine Genome Array, CA, USA	To study the effects of metabolic regulators on blastocysts	[150]
	To test the effects of different culture media on IVP embryos	[151]
	To study transcriptomic dynamics at EGA (8-cell stage)	[152]
	To identify stage-specific expression patterns of in vivo developing 2-cell, 4-cell, and 8-cell stages and of morulae and blastocysts	[153]
	To study effects of elevated progesterone on blastocyst development	[154]
	To compare degenerated and healthy-looking IVP blastocysts	[155]
	To study sex-specific gene expression patterns of blastocysts	[156]
	To identify transcriptome fingerprints as predictors of pregnancy success after ET	[157]
	To study the effects of superovulation on embryo development	[158]
	To study the transition of a spherical blastocyst to an ovoid conceptus	[159]
	To study the effects of metabolic regulators on blastocysts	[150]
	To study cell lineage specification of blastocysts	[160]
EmbryoGENE Bovine Microarray, Agilent, CA, USA	To study EGA and effects of in vivo and in vitro culture conditions on early embryo development	[161]
	To study the effects of hyperglycemic stress on IVP blastocysts	[162]
	To study the effects of oxidative stress on IVP blastocysts	[163]
	To compare the ICM and TE between IVP and IVD embryos	[164]
	To study the effects of non-esterified fatty acid (NEFA) concentrations on blastocysts	[165]
	To compare effects of vitrification and slow freezing on morulae and blastocysts	[166]
	To study effects of bull age on blastocysts	[167]
	To identify transcriptomic predictors of pregnancy outcomes	[168]
	To study effects of perfluorooctane sulfonate (PFOS) exposure of bovine oocytes on early embryonic development	[169]
	To model a gene signature predictive of embryonic survival	[170]
Bovine gene expression microarray V2, Agilent, CA, USA	To study the transcriptomic profile related to bovine pluripotency	[171]
3995 bovine cDNA microarray developed in-house	To study temporal changes during peri-implantation developmental stages (7–28-day-old embryos)	[172]
7872 bovine cDNA microarray developed by University of Illinois, Urbana-Champaign	To compare IVD and somatic cell nuclear transfer (SCNT)-derived blastocysts	[173]
2640 bovine cDNA microarray developed in-house	To compare IVP- and SCNT-derived blastocysts	[174]
5000 bovine cDNA microarray developed in-house	To compare IVP- and SCNT-derived blastocysts	[175]

**Table 8:** Applications of RNA-sequencing technology in bovine embryo evaluation and analysis.

RNA-Seq Platform Used	Objective/s of the Study	Source
Genome Analyzer, Illumina, CA, USA	To compare healthy-looking and degenerated IVP blastocysts	[178]
	To study maternal recognition and implantation using 7-, 10-, 13-, 16-, and 19-day-old bovine embryos	[179]
	To study EGA using 4-cell, 8-cell, 16-cell, and blastocyst stages	[180]
	To demonstrate that RNA-seq can be carried out using RNA extracted from individual blastocysts	[181]
	To identify microRNA (miRNA) in culture media of embryos of differing developmental competence	[187]
HiSeq 1500^TM^ system, Illumina, CA, USA	To study the function of the lineage-specific gene *OCT4* (by knocking it out using CRISPR/Cas-9 system)	[79]
	To study EGA using single-blastomere RNA-seq	[186]
	To study the role of *OCT4* in the second lineage differentiation	[188]
HiSeq 2000^TM^ system, Illumina, CA, USA	To compare morphologically similar IVD and IVP blastocysts	[25]
	To demonstrate that RNA-seq can be carried out using RNA extracted from single ICM and TE cell “biopsies”	[189]
	To study the paternal genetic contribution on pre-implantation IVP blastocysts	[190]
HiSeq 2500^TM^ system, Illumina, CA, USA	To study miRNA expression in zygotes	[191]
HiSeq 3000^TM^ system, Illumina, CA, USA	To test the effects of DKK1 (WNT antagonist) during the morula and blastocyst stages	[192]
HiSeq 4000^TM^ system, Illumina, CA, USA	To study the paternal genetic contribution in pre-implantation IVD blastocysts	[193]
NextSeq^TM^ system, Illumina, CA, USA	To identify transcriptomic signatures predictive of establishing and maintaining gestation	[183,184]
	To study chromatin remodeling events during EGA	[194]
HiSeq X Ten^TM^ system, Illumina, CA, USA	To study paternally contributed RNAs (sperm-derived RNAs) in pre-EGA (2-cell and 4-cell) embryos	[195]
	To determine the role of the chromatin remodeler SMARCA5 in blastocysts	[196]
	To determine the role of the NOTCH signaling pathway in early embryonic development	[197]
	To study the spatiotemporal translational regulation during pre-implantation development	[198]
NovaSeq6000^TM^ system, Illumina, CA, USA	To study lipid-metabolism-associated gene expression in parthenogenetic embryos	[199]
	To study the functional consequences of three critical lineage-specific genes (*SOX2*, *OCT4*, and *CDX2*)	[182]
	sexing of embryos	[200]
5500xl Genetic Analyzer, Applied Biosystems, CA, USA	To compare 2-cell to 16-cell embryos and early morulae and blastocysts to human and mice embryos	[201]
	To quantify transcript abundance of imprinted genes	[202]
	To detect embryo developmental potential by single-blastomere RNA-seq	[185]

**Table 10:** Miscellaneous tools/techniques used to analyze bovine embryos.

Marker Measured/Methodology	Equipment/Methodology Used	Source/s
Apoptosis	TUNEL assay	[71,239,240], reviewed by [241]
Ribosome profile	High-resolution ribosome fractionation, polysome profiling, RNA-seq	[198]
DNA methylation landscape	Anti-5-methylcytosine (5-MeC) antibody, methylation-sensitive high-resolution melting analysis (MS-HRM), bisulfite sequencing	[75,242,243,244,245,246,247,248]
mRNA localization	In situ hybridization	[63,80,249,250]
Chromosomal abnormalities	5% Giemsa staining (1250× magnification)	[251]
Automation/artificial intelligence (AI)-based microscopy	Use of genetic algorithms, artificial neural networks, automatic feature extraction from images and supervised learning to formulate AI-based computer-assisted scoring systems (CASS)/predictive models	[1,252,253,254,255,256]
Gene knockdown	Cytosine base editing, CRISPR/Cas-9 gene editing, siRNA-mediated gene knockdown	[79,182,196,197,257]
Characterization of extracellular vesicles	Nanoparticle tracking analysis, electron microscopy, small RNA sequencing	[258,259,260]
Multi-omics approaches	Integrating transcriptomic and epigenetic data and integrating metabolomics and epigenetic data	[261,262]

**Table 11:** Comparison of the different tools available for embryo evaluation (green indicates a favorable feature, whereas orange indicates an unfavorable feature).

	Accuracy	Invasive Nature	Subjectivity	Initial Cost and/or Cost of Operation	Technical Complexity	Evaluation Complete by Blastulation	Need Extra Freezing Step
Stereoscopy	Low	Low	High	Low	Low	No	No
Fluorescence microscopy	High	High	Low	High	High	N/A **	N/A **
Electron microscopy	High	High	Low	High	High	N/A **	N/A **
GLIM	High	Low	Low	High	High	No	No
TLM	High	Low	Low	High	Low	Yes	No
AI-based microscopy	High	Low	Low	Low	High	No	No
Genomics	High	High *	Low	High	High	No	Yes
Transcriptomics	High	High *	Low	High	High	No	Yes
Proteomics	High	Low	Low	High	High	No	Yes
Metabolomics	High	Low	Low	High	High	No	Yes
NMR	High	Low	Low	High	High	No	Yes

* If blastomere biopsies are tested instead of whole embryos, embryos will still be viable and can be used for ET post-testing, but it is nevertheless considered invasive. ** Embryos lose viability before/during evaluation, and therefore, these criteria are non-applicable.

## Data Availability

As this is a review article, all data used in the preparation of this manuscript are freely available to the public.

## Supplementary Materials

The following supporting information can be downloaded at https://www.mdpi.com/article/10.3390/ani13132102/s1: Figures S1–S19. Figure S1. 100× DIC. Stage 5 Grade 1 embryo. Early blastocyst collected day 7 post onset of estrus. Notice the presence of blastomeres immediately inside the VM causing an irregular bulge, or bumpy edge to the VM. The inner cell mass plus the blastocoele and VM is collectively called the embryo proper. Compare the bulges on the VM of this early blastocyst to the very smooth non-bulging VM of the unfertilized ova (UFO). *[Figure and caption provided with permission of the International Embryo Technology Society (IETS) 2023].* Figure S2. 100× DIC. Same embryo as slide #4 and #5, but arrows point to individual blastomeres. Also, one fused blastomere is outlined in yellow*[Figure and caption provided with permission of the International Embryo Technology Society (IETS) 2023]*. Figure S3. 400× DIC. Stage 4, Grade 1. This is an anatomically near perfect day 7 morula. There are very few, if any, extruded blastomeres outside the VM. The differentiating factor between this and a UFO is the bulging of the blastomeres in this embryo causing the VM to be irregular, yet contiguous and visible. Close inspection of the embryo proper allows one to see a large mass of fused blastomeres. *[Figure and caption provided with permission of the International Embryo Technology Society (IETS) 2023].* Figure S4. 400× BF. Stage 4, Grade 2. Arguably, this embryo could be considered a grade 1 instead of a grade 2. The main mass (circled - see next slide) has a very well defined VM covering a healthy group of well fused blastomeres. In the visible plane there are two large extruded blastomeres. Rolling the embryo revealed two more smaller ones on the back side. This is a good example of the subjectivity involved in grading embryos. *[Figure and caption provided with permission of the International Embryo Technology Society (IETS) 2023].* Figure S5. 100× DIC. Stage 4, Grade 3. This morula has several extruded unfused cells (arrows) in this plane, plus a few others as it was observed while rolling. The healthy fused mass in the middle represents about half of the total blastomeres. *[Figure and caption provided with permission of the International Embryo Technology Society (IETS) 2023].* Figure S6. 200× BF. Stage 5 Grade 1. The arrow points to the "hollow" early developing blastocoele cavity. *[Figure and caption provided with permission of the International Embryo Technology Society (IETS) 2023].* Figure S7. 400× DIC. Stage 5, Grade 3. The viable cells in this embryo have differentiated and formed a blastocoele (lower left part of encircled cell mass–see next slide–hollow cavity). The remainder of the cells are unfused and dead. *[Figure and caption provided with permission of the International Embryo Technology Society (IETS) 2023].* Figure S8. 200× DIC. Stage 6, Grade 1. The yellow arrow points to the blastocoele cavity. The lighter colored blastocoele has slightly more total volume than the inner cell mass. That is the standard for classifying the embryo as a late-stage blastocyst (stage 6). The blue arrow points to the trophoblast. The red arrow points to the inner cell mass. *[Figure and caption provided with permission of the International Embryo Technology Society (IETS) 2023].* Figure S9. 100× DIC. Stage 6, Grade 1. The blastocoele is about twice the size of the inner cell mass. The embryo proper has grown to the point that the VM now contacts the zona, thus eliminating a visible perivitelline space. However, the zona has not begun to expand geometrically and thin, thus this embryo is classified as a Stage 6 instead of Stage 7 (expanded blastocyst). One important point should be noted about embryos that have no perivitelline space–many of these embryos will be classified as Grade 1’s because any extruded blastomeres are flattened between the VM and the zona, and are, therefore often difficult to visualize. *[Figure and caption provided with permission of the International Embryo Technology Society (IETS) 2023].* Figure S10. 100× DIC. Stage 6, Grade 2. Although the embryo proper has no imperfections, the zona is severely cracked and torn at 9 o’clock resulting in a down Grade from a 1 to a 2. This embryo would not qualify for export. *[Figure and caption provided with permission of the International Embryo Technology Society (IETS) 2023].* Figure S11. 100× DIC. Stage 6 (arguably a stage 5), Grade 3. When this embryo was rolled during evaluation it was easier to see that the blastocoele (circle–see next slide) was slightly larger than the inner cell mass. Also, about half of the total cell mass was dead making the embryo a Grade 3. *[Figure and caption provided with permission of the International Embryo Technology Society (IETS) 2023].* Figure S12. 100× DIC. Stage 7, Grade 1. The blastocoele represents about 70% of the embryo proper, and the inner cell mass (yellow arrow) is relatively smaller (about 30% of the embryo proper). The embryo proper has expanded all the way to the zona, and the zona is stretching/thinning to accommodate the growth. A thin layer of trophoblast extends from the corners of the inner cell mass and extends along the entire outer border of the blastocoele and approximates the inner border of the zona. *[Figure and caption provided with permission of the International Embryo Technology Society (IETS) 2023].* Figure S13. 400× BF. Hatching blastocyst, Stage 8, Grade 1. The zona has cracked (6 to 8 o’clock) under the pressure of growth by the blastocyst. *[Figure and caption provided with permission of the International Embryo Technology Society (IETS) 2023].* Figure S14. 400× BF. Stage 8, Grade 1. This is a hatched blastocyst in almost perfect condition. Normally, the ratio of blastocoele to inner cell mass would be greater than in this embryo. *[Figure and caption provided with permission of the International Embryo Technology Society (IETS) 2023].* Figure S15. 100× DIC. #1 is an empty zona. See tear at 9 o’clock. Notice how the zona wall is thinner in #1 as compared to #2 and #3 (both stage 7, grade 1). #4 is a hatched blastocyst (Stage 8, Grade 1) without a zona. #1 is likely the zona that belongs to #4. *[Figure and caption provided with permission of the International Embryo Technology Society (IETS) 2023].* Figure S16. 100× DIC. The three embryos with yellow arrows are Stage 7, Grade 2s. Notice the brown colored granular appearance of the blastocoele cavities of those embryos compared to the expanded blastocysts with translucent (green arrows) in this microphotograph. The granulation is an artifact of cellular debris along with dead and degenerated cells trapped tightly between the trophoblast and the zona. The green arrows point to Grade 1 embryos. *[Figure and caption provided with permission of the International Embryo Technology Society (IETS) 2023].* Figure S17. 100× DIC. #1 = Stage 7, Grade 1 (7-1). #2 = 6-1, #3 = 5-1, #4 = 6-1. #5 = 6-1, #6 = 6-1,. #7 = 7-1 (notice diameter compared to bordering embryos), #8 = 5-1. *[Figure and caption provided with permission of the International Embryo Technology Society (IETS) 2023].* Figure S18. 40× BF. The red arrows point to Stage 7, Grade 1 embryos. The green arrows point to Stage 6, Grade 1 embryos. The yellow arrows represent Stage 5, Grade 1 embryos. The blue arrow points to Stage 4, Grade 1s. The orange dashed arrows point to fragmented UFOs. Any embryo with a cracked zona downgrades from a Grade 1 to a Grade 2. *[Figure and caption provided with permission of the International Embryo Technology Society (IETS) 2023].* Figure S19. 40× BF. #1 = Stage 6, Grade 1. #2 and #3 = Stage 7, Grade 1. #4 - Stage 6, Grade 1. The blastocoele appears to be smaller than the inner cell mass (ICM) in this view, but when rolled, the blastocoele is larger than the ICM. #5 = Stage 7, Grade 1. Notice the large diameter, plus the thinning of zona due to physical expansion of growth. #6 = Stage 7, Grade 1 (arguably a stage 6). #7 = Stage 6, Grade 1 (arguably a stage 5). #8 = Stage 7, Grade 1. #9 and #10 = Stage 5, Grade 1. #11 and #12 = Stage 7, Grade 1. #13 = Stage 5, Grade 1 (arguably a stage 6). #14 = Stage 4, Grade 2 (arguably a grade 1). 15 = Stage 5, Grade 1. #16 = Stage 7, Grade 1. #17 = Stage 4, Grade 3. The VM is very ill defined in this embryo. The VM has a "frayed" appearance from about 12 to 6 o’clock. There are most likely a few viable fused cells in the main mass, but many of the peripheral cells are degenerated. #18 = Stage 4, Grade 2. It has dead blastomeres at 6, 7, and 12 o’clock. #19 = Stage 5, Grade 1 (arguably a stage 6). #20 = Stage 6, Grade 1. #21 = Stage 7, Grade 1 (arguably stage 6). #22 = Stage 5, Grade 1. #23, 24, and 25 = Stage 4, Grade 1. #26 = Stage 4, Grade 2. The shape of the embryo proper is geometrically non-spherical. #27 = Stage 4, Grade 2 (arguably a stage 5) due to a cracked zona at 3 o’clock. #28 = Stage 7, Grade 1. #29 = Stage 6, Grade 1. This embryo is slightly oval shaped, but has no other flaws. #30 = Stage 7, Grade 1. #31 = Stage 6, Grade 1. #32 = Stage 7, Grade 1. #33 = Stage 6, Grade 1. #34 = Stage 4, Grade 1. #35 = Stage 7, Grade 1. #36 = Stage 7, Grade 1 (arguably stage 6). #37 = Stage 4, Grade 2 (cracked zona). This embryo could be a Stage 7 with a collapsed blastocoele cavity due to physical forces during the embryo collection procedure. The diameter of the zona is noticeably larger than that of embryo 34. The crack in the zona is at about 7 o’clock. #38 = Stage 7, Grade 1. #39 = Stage 5, Grade 1. #40 = Stage 6, Grade 1. *[Figure and caption provided with permission of the International Embryo Technology Society (IETS) 2023].*
