# Supplementary material for: Pre-Implantation Bovine Embryo Evaluation—From Optics to Omics and Beyond
Source: Animals (Basel). 2023 Jun 24;13(13):2102. doi: 10.3390/ani13132102 (PMC10339960; doi:10.3390/ani13132102)
Supplement: Supplementary file 1 [file animals-13-02102-s001.zip › animals-2438292-supplementary.pdf]

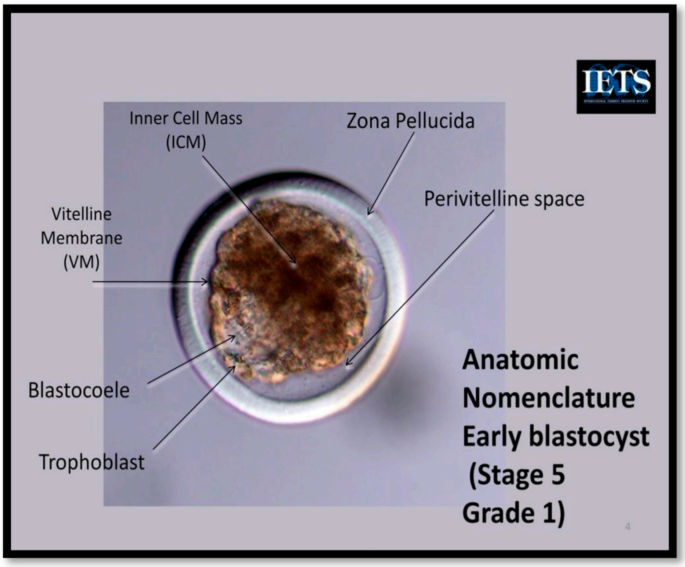

Figure S1. 100x DIC. Stage 5 Grade 1 embryo. Early blastocyst collected day 7 post onset of estrus. Notice the presence of blastomeres immediately inside the VM causing an irregular bulge, or bumpy edge to the VM. The inner cell mass plus the blastocoele and VM is collectively called the embryo proper. Compare the bulges on the VM of this early blastocyst to the very smooth non-bulging VM of the unfertilized ova (UFO). *[Figure and caption provided with permission of the International Embryo Technology Society (IETS) 2023].*

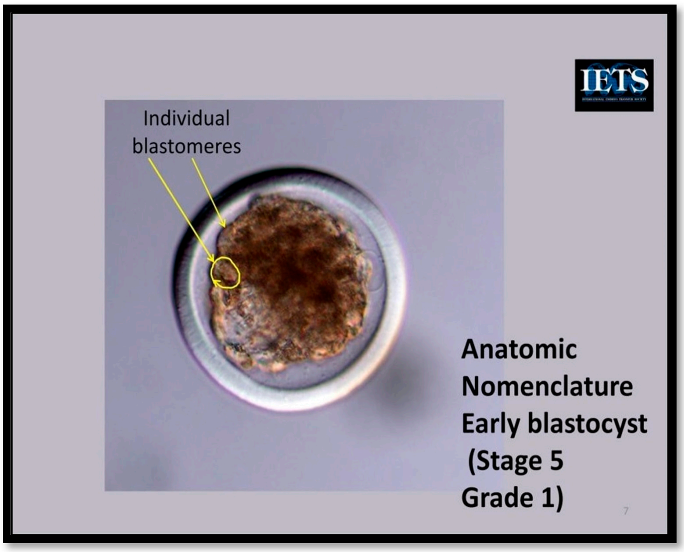

Figure S2. 100x DIC. Same embryo as slide #4 and #5, but arrows point to individual blastomeres. Also, one fused blastomere is outlined in yellow*[Figure and caption provided with permission of the International Embryo Technology Society (IETS) 2023].*

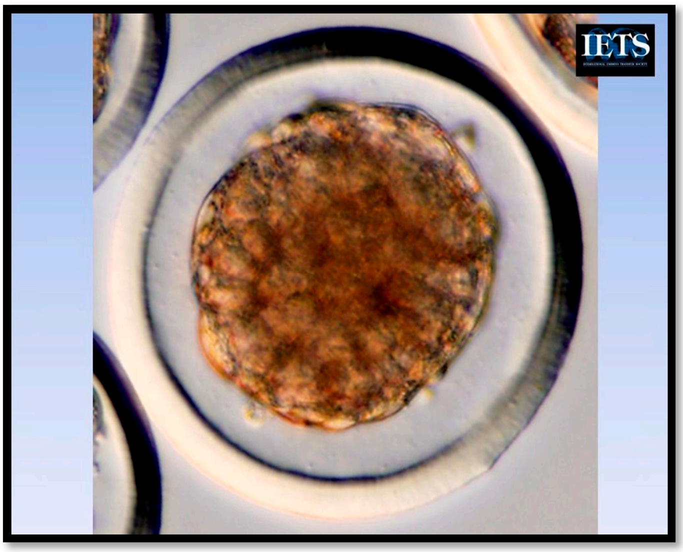

Figure S3. 400x DIC. Stage 4, Grade 1. This is an anatomically near perfect day 7 morula. There are very few, if any, extruded blastomeres outside the VM. The differentiating factor between this and a UFO is the bulging of the blastomeres in this embryo causing the VM to be irregular, yet contiguous and visible. Close inspection of the embryo proper allows one to see a large mass of fused blastomeres. *[Figure and caption provided with permission of the International Embryo Technology Society (IETS) 2023].*

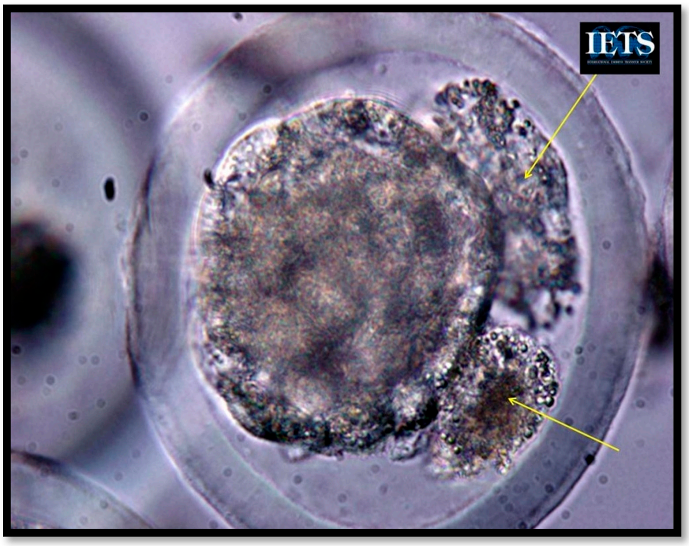

Figure S4. 400x BF. Stage 4, Grade 2. Arguably, this embryo could be considered a grade 1 instead of a grade 2. The main mass (circled - see next slide) has a very well defined VM covering a healthy group of well fused blastomeres. In the visible plane there are two large extruded blastomeres. Rolling the embryo revealed two more smaller ones on the back side. This is a good example of the subjectivity involved in grading embryos. *[Figure and caption provided with permission of the International Embryo Technology Society (IETS) 2023].*

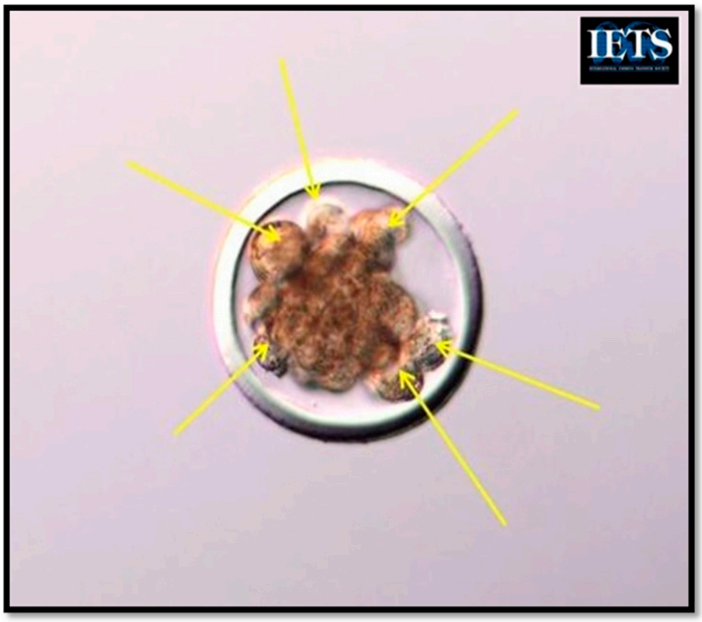

Figure S5. 100x DIC. Stage 4, Grade 3. This morula has several extruded unfused cells (arrows) in this plane, plus a few others as it was observed while rolling. The healthy fused mass in the middle represents about half of the total blastomeres. *[Figure and caption provided with permission of the International Embryo Technology Society (IETS) 2023].*

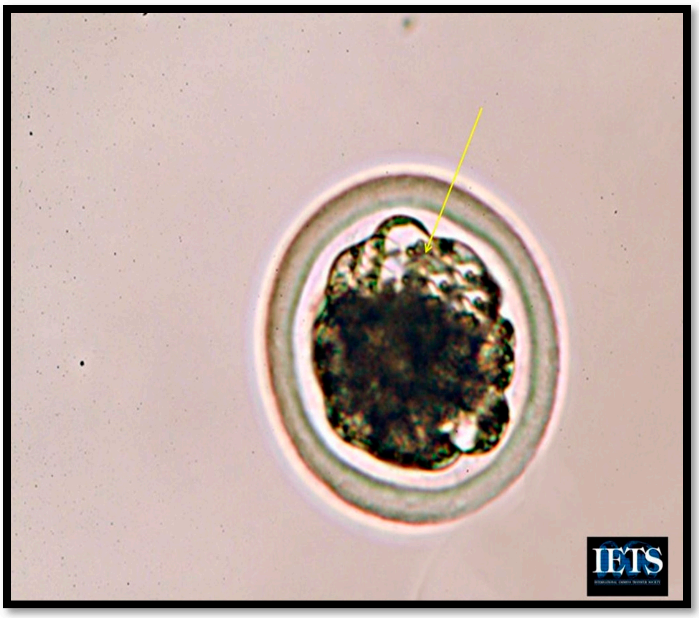

Figure S6. 200x BF. Stage 5 Grade 1. The arrow points to the "hollow" early developing blastocoele cavity. *[Figure and caption provided with permission of the International Embryo Technology Society (IETS) 2023].*

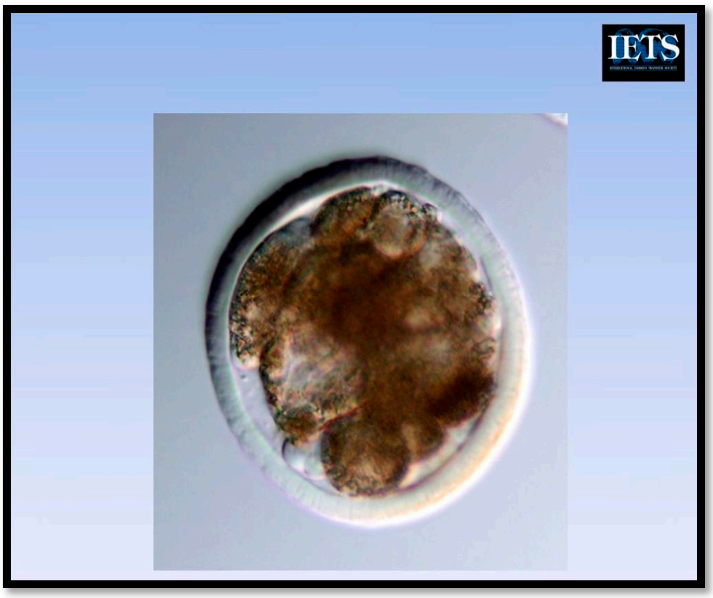

Figure S7. 400x DIC. Stage 5, Grade 3. The viable cells in this embryo have differentiated and formed a blastocoele (lower left part of encircled cell mass – see next slide – hollow cavity). The remainder of the cells are unfused and dead. *[Figure and caption provided with permission of the International Embryo Technology Society (IETS) 2023].*

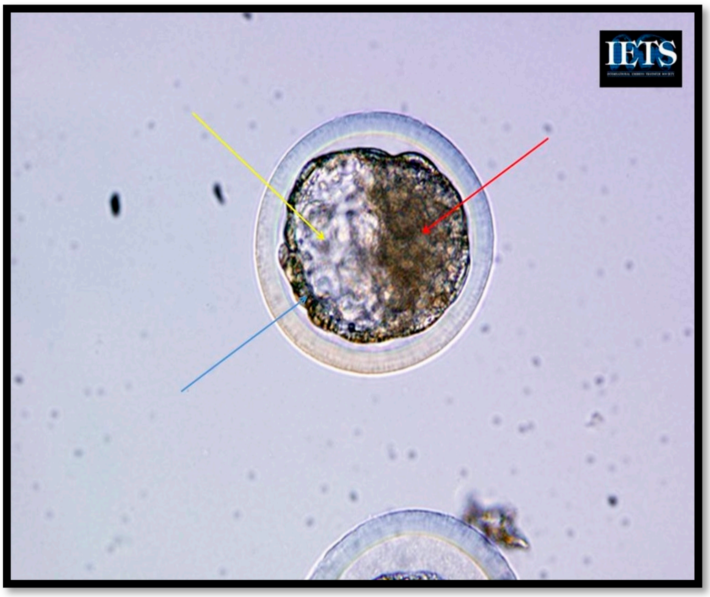

Figure S8. 200x DIC. Stage 6, Grade 1. The yellow arrow points to the blastocoele cavity. The lighter colored blastocoele has slightly more total volume than the inner cell mass. That is the standard for classifying the embryo as a late-stage blastocyst (stage 6). The blue arrow points to the trophoblast. The red arrow points to the inner cell mass. *[Figure and caption provided with permission of the International Embryo Technology Society (IETS) 2023].*

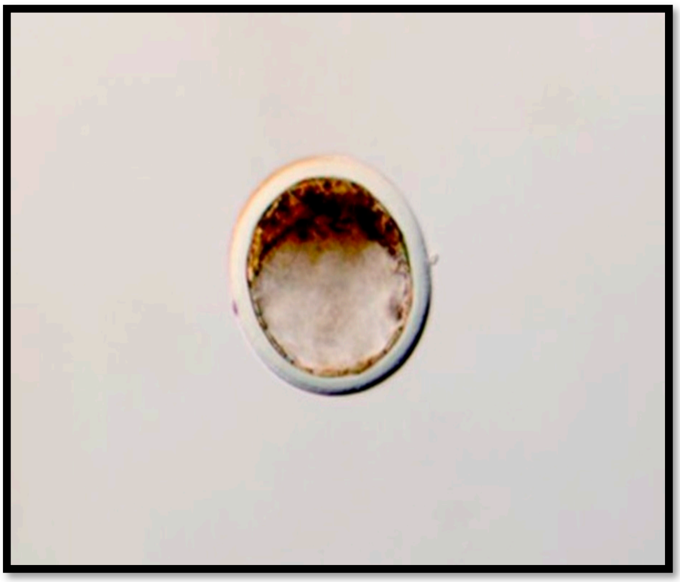

Figure S9. 100x DIC. Stage 6, Grade 1. The blastocoele is about twice the size of the inner cell mass. The embryo proper has grown to the point that the VM now contacts the zona, thus eliminating a visible perivitelline space. However, the zona has not begun to expand geometrically and thin, thus this embryo is classified as a Stage 6 instead of Stage 7 (expanded blastocyst). One important point should be noted about embryos that have no perivitelline space – many of these embryos will be classified as Grade 1's because any extruded blastomeres are flattened between the VM and the zona, and are, therefore often difficult to visualize. *[Figure and caption provided with permission of the International Embryo Technology Society (IETS) 2023].*

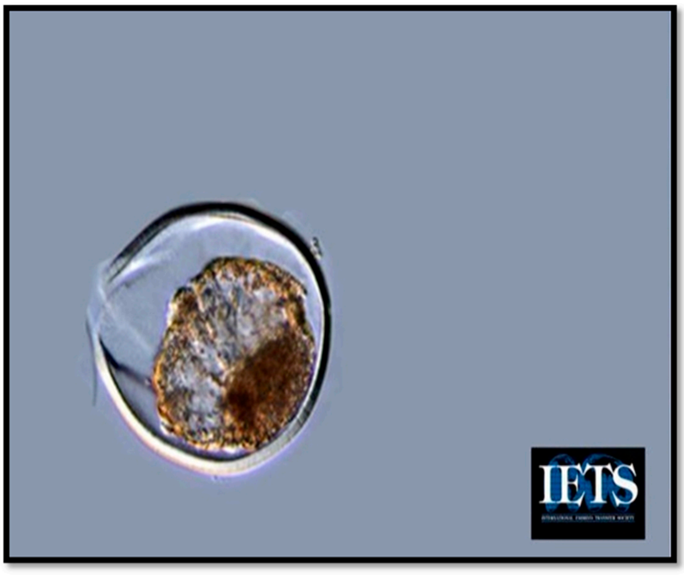

Figure S10. 100x DIC. Stage 6, Grade 2. Although the embryo proper has no imperfections, the zona is severely cracked and torn at 9 o'clock resulting in a down Grade from a 1 to a 2. This embryo would not qualify for export. *[Figure and caption provided with permission of the International Embryo Technology Society (IETS) 2023].*

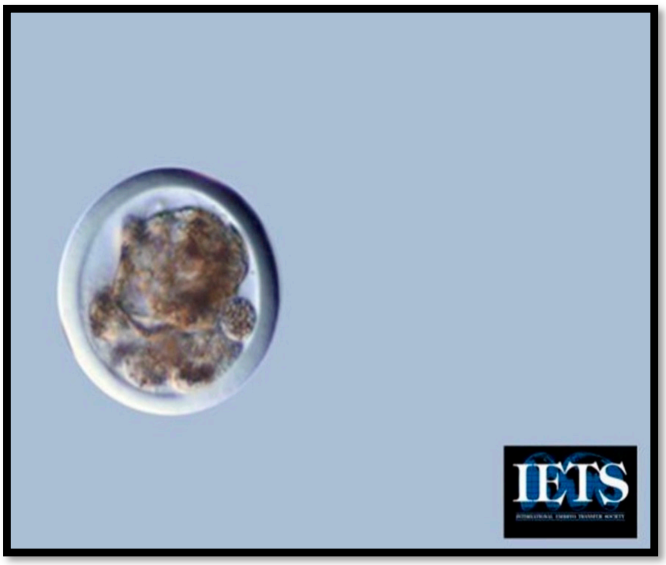

Figure S11. 100x DIC. Stage 6 (arguably a stage 5), Grade 3. When this embryo was rolled during evaluation it was easier to see that the blastocoele (circle – see next slide) was slightly larger than the inner cell mass. Also, about half of the total cell mass was dead making the embryo a Grade 3. *[Figure and caption provided with permission of the International Embryo Technology Society (IETS) 2023].*

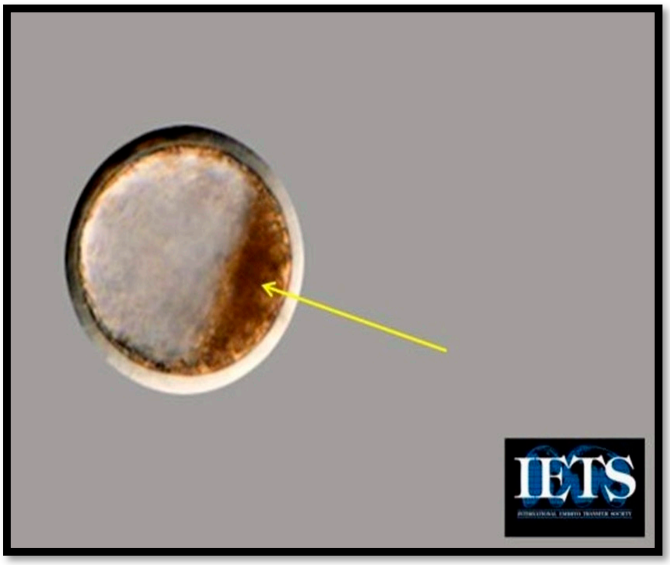

Figure S12. 100x DIC. Stage 7, Grade 1. The blastocoele represents about 70% of the embryo proper, and the inner cell mass (yellow arrow) is relatively smaller (about 30% of the embryo proper). The embryo proper has expanded all the way to the zona, and the zona is stretching/thinning to accommodate the growth. A thin layer of trophoblast extends from the corners of the inner cell mass and extends along the entire outer border of the blastocoele and approximates the inner border of the zona. *[Figure and caption provided with permission of the International Embryo Technology Society (IETS) 2023].*

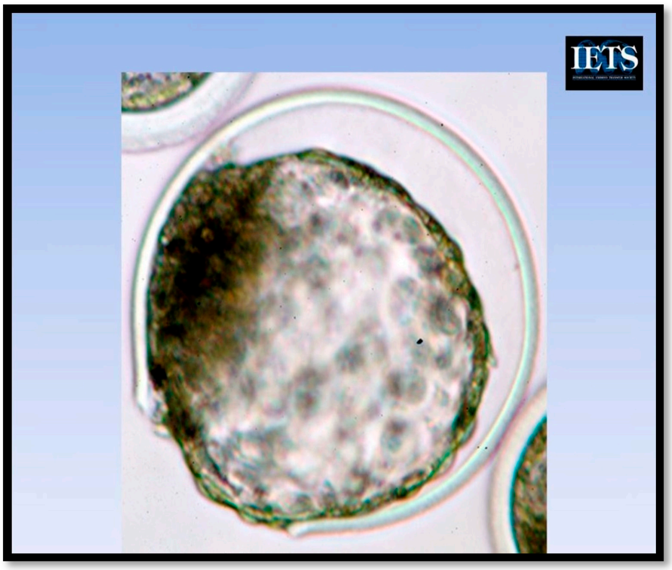

Figure S13. 400x BF. Hatching blastocyst, Stage 8, Grade 1. The zona has cracked (6 to 8 o'clock) under the pressure of growth by the blastocyst. *[Figure and caption provided with permission of the International Embryo Technology Society (IETS) 2023].*

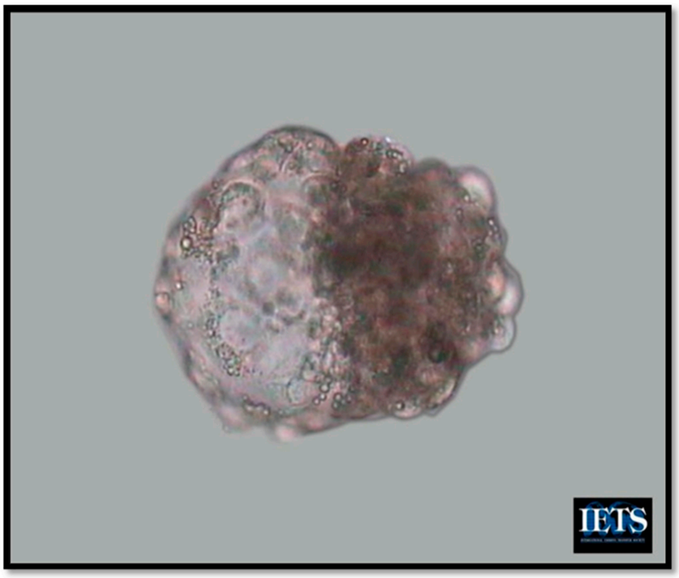

Figure S14. 400x BF. Stage 8, Grade 1. This is a hatched blastocyst in almost perfect condition. Normally, the ratio of blastocoele to inner cell mass would be greater than in this embryo. *[Figure and caption provided with permission of the International Embryo Technology Society (IETS) 2023].*

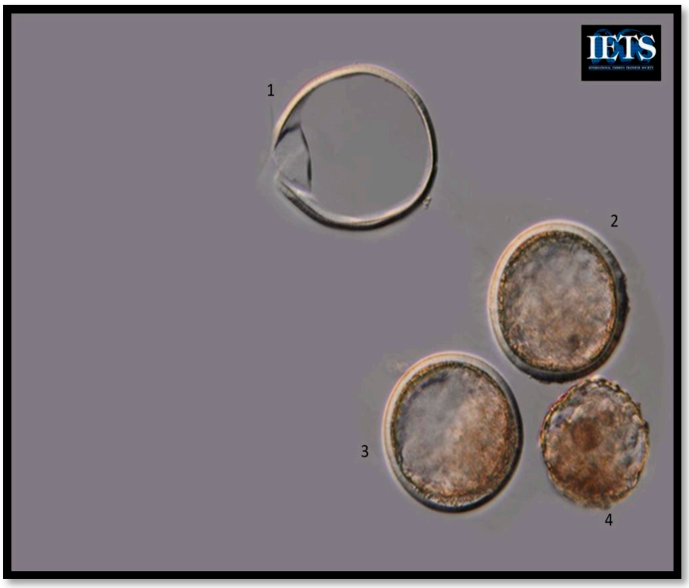

Figure S15. 100x DIC. #1 is an empty zona. See tear at 9 o'clock. Notice how the zona wall is thinner in #1 as compared to #2 and #3 (both stage 7, grade 1). #4 is a hatched blastocyst (Stage 8, Grade 1) without a zona. #1 is likely the zona that belongs to #4. *[Figure and caption provided with permission of the International Embryo Technology Society (IETS) 2023].*

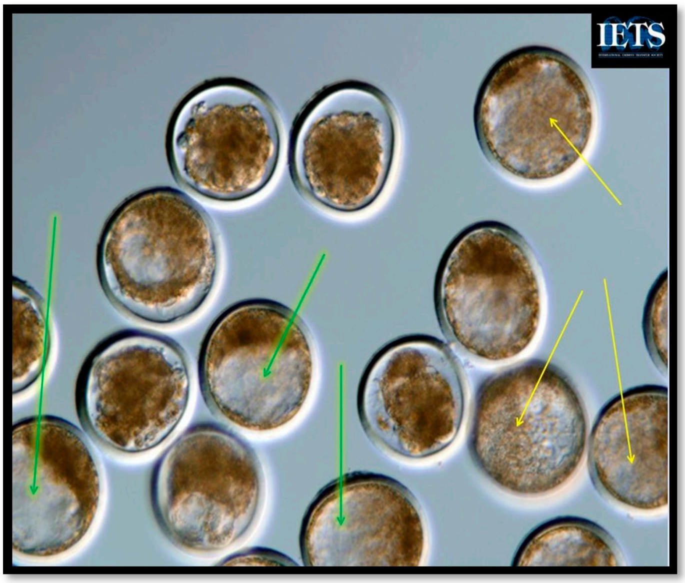

Figure S16. 100x DIC. The three embryos with yellow arrows are Stage 7, Grade 2s. Notice the brown colored granular appearance of the blastocoele cavities of those embryos compared to the expanded blastocysts with translucent (green arrows) in this microphotograph. The granulation is an artifact of cellular debris along with dead and degenerated cells trapped tightly between the trophoblast and the zona. The green arrows point to Grade 1 embryos. *[Figure and caption provided with permission of the International Embryo Technology Society (IETS) 2023].*

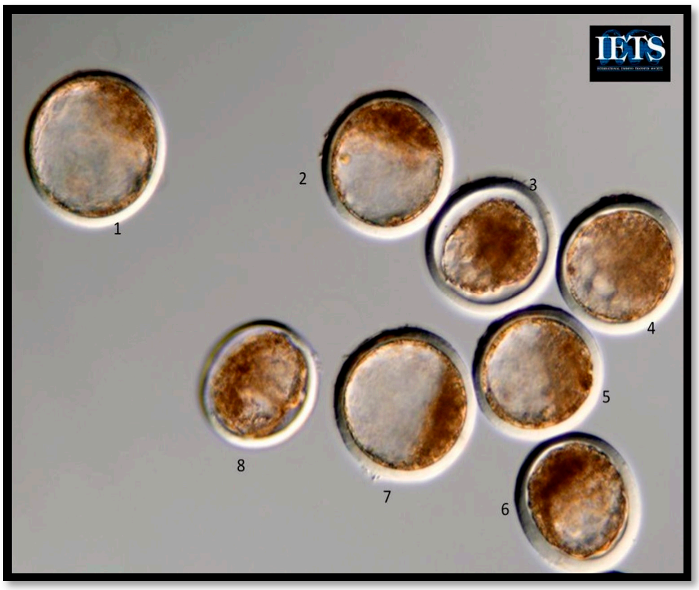

Figure S17. 100x DIC. #1 = Stage 7, Grade 1 (7-1). #2 = 6-1, #3 = 5-1, #4 = 6-1. #5 = 6-1, #6 = 6-1, #7 = 7-1 (notice diameter compared to bordering embryos), #8 = 5-1. *[Figure and caption provided with permission of the International Embryo Technology Society (IETS) 2023].*

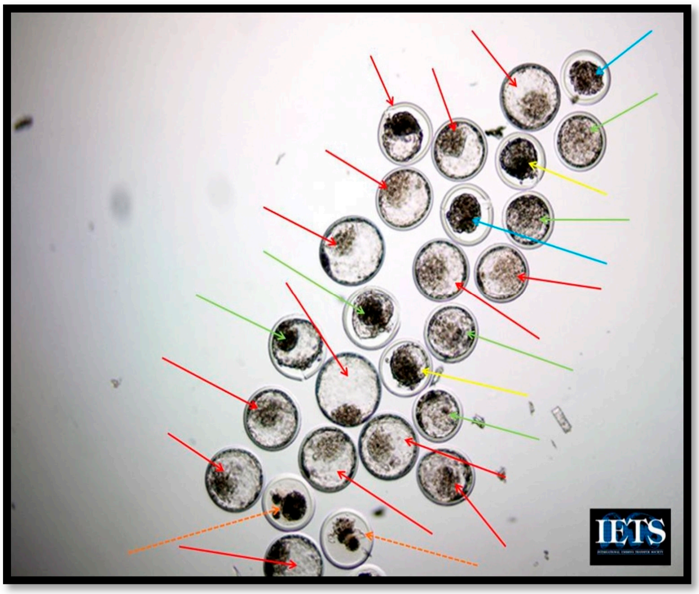

Figure S18. 40x BF. The red arrows point to Stage 7, Grade 1 embryos. The green arrows point to Stage 6, Grade 1 embryos. The yellow arrows represent Stage 5, Grade 1 embryos. The blue arrow points to Stage 4, Grade 1s. The orange dashed arrows point to fragmented UFOs. Any embryo with a cracked zona downgrades from a Grade 1 to a Grade 2. *[Figure and caption provided with permission of the International Embryo Technology Society (IETS) 2023].*

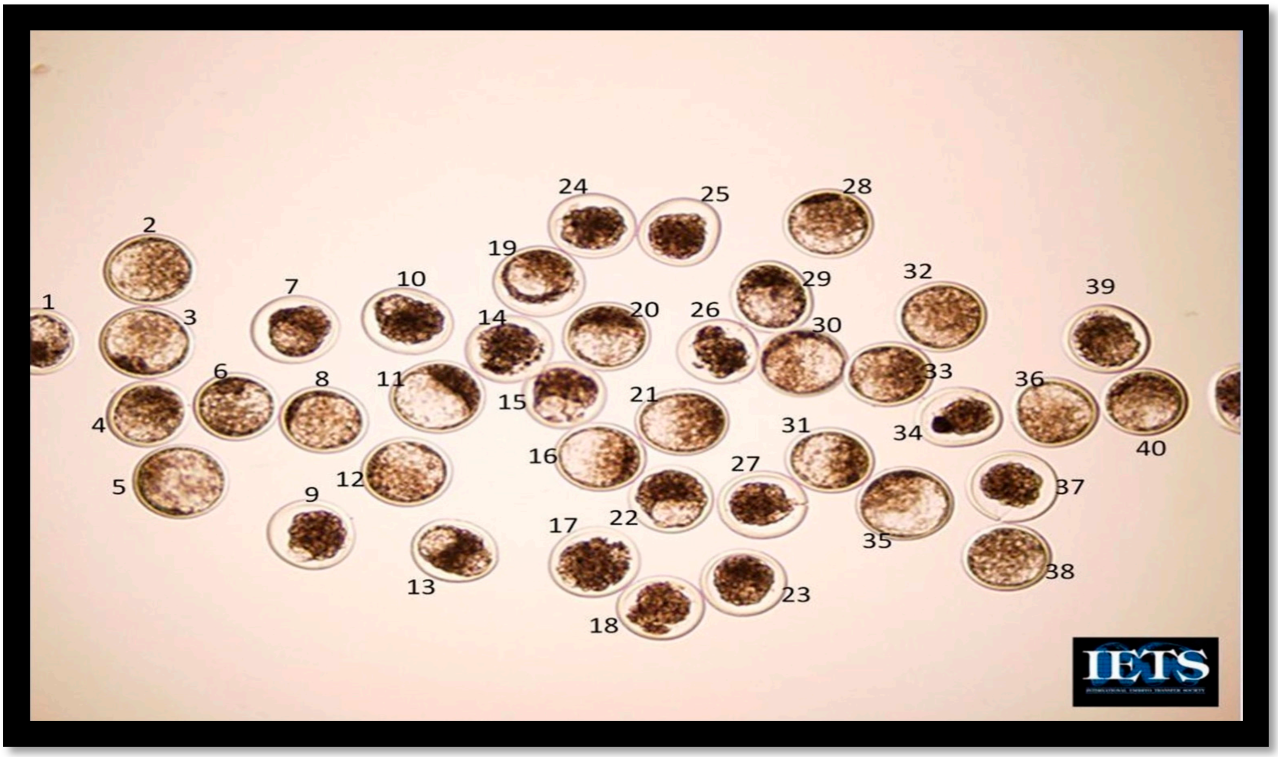

Figure S19. 40x BF. #1 = Stage 6, Grade 1. #2 and #3 = Stage 7, Grade 1. #4 - Stage 6, Grade 1. The blastocoele appears to be smaller than the inner cell mass (ICM) in this view, but when rolled, the blastocoele is larger than the ICM. #5 = Stage 7, Grade 1. Notice the large diameter, plus the thinning of zona due to physical expansion of growth. #6 = Stage 7, Grade 1 (arguably a stage 6). #7 = Stage 6, Grade 1 (arguably a stage 5). #8 = Stage 7, Grade 1. #9 and #10 = Stage 5, Grade 1. #11 and #12 = Stage 7, Grade 1. #13 = Stage 5, Grade 1 (arguably a stage 6). #14 = Stage 4, Grade 2 (arguably a grade 1). 15 = Stage 5, Grade 1. #16 = Stage 7, Grade 1. #17 = Stage 4, Grade 3. The VM is very ill defined in this embryo. The VM has a "frayed" appearance from about 12 to 6 o'clock. There are most likely a few viable fused cells in the main mass, but many of the peripheral cells are degenerated. #18 = Stage 4, Grade 2. It has dead blastomeres at 6, 7, and 12 o'clock. #19 = Stage 5, Grade 1 (arguably a stage 6). #20 = Stage 6, Grade 1. #21 = Stage 7, Grade 1 (arguably stage 6). #22 = Stage 5, Grade 1. #23, 24, and 25 = Stage 4, Grade 1. #26 = Stage 4, Grade 2. The shape of the embryo proper is geometrically non-spherical. #27 = Stage 4, Grade 2 (arguably a stage 5) due to a cracked zona at 3 o'clock. #28 = Stage 7, Grade 1. #29 = Stage 6, Grade 1. This embryo is slightly oval shaped, but has no other flaws. #30 = Stage 7, Grade 1. #31 = Stage 6, Grade 1. #32 = Stage 7, Grade 1. #33 = Stage 6, Grade 1. #34 = Stage 4, Grade 1. #35 = Stage 7, Grade 1. #36 = Stage 7, Grade 1 (arguably stage 6). #37 = Stage 4, Grade 2 (cracked zona). This embryo could be a Stage 7 with a collapsed blastocoele cavity due to physical forces during the embryo collection procedure. The diameter of the zona is noticeably larger than that of embryo 34. The crack in the zona is at about 7 o'clock. #38 = Stage 7, Grade 1. #39 = Stage 5, Grade 1. #40 = Stage 6, Grade 1. *[Figure and caption provided with permission of the International Embryo Technology Society (IETS) 2023].*
